# 3D-Printed Microfiltration Membranes via Dual-Wavelength Microstereolithography

**DOI:** 10.1021/acsomega.5c05746

**Published:** 2025-08-21

**Authors:** Hanieh Bazyar, Shang-Che Wu, Irem Gurbuz, Athanasios Papageorgiou, Wesley van Vliet, Alexander Kostenko, Jimmy G. Jean, Guillaume Broggi, Baris Caglar

**Affiliations:** † Transport Phenomena, Chemical Engineering Department, Faculty of Applied Sciences, 2860 Delft University of Technology, Van der Maasweg 9, Delft 2629HZ, The Netherlands; ‡ Engineering Thermodynamics, Process & Energy Department, Faculty of Mechanical Engineering, Delft University of Technology, Leeghwaterstraat 39, Delft 2628CB, The Netherlands; § Photosynthetic B.V., De Boelelaan 1085, Amsterdam 1081HV, The Netherlands; ∥ Aerospace Structures and Materials Department, Faculty of Aerospace Engineering, Delft University of Technology, Kluyverweg 1, Delft 2629HS, The Netherlands

## Abstract

A new and sustainable membrane manufacturing method is 3D printing, which reduces the number of fabrication steps, waste production, and the corresponding CO_2_ emissions. It further enables fabricating membranes with well-defined pore size, shape, and configuration. Here, we study 3D printing of microfiltration membranes using a novel dual-wavelength microstereolithography method. Via the gradient descent method, we are able to calculate and control a printable membrane with micrometer precision, enabling the possibility of printing membranes directly. Hydrophilic porous membranes with cylindrical microscale pores (≈10 μm in diameter) are printed from polyethylene glycol diacrylate (PEGDA). Membrane printing procedure and postprocessing steps are thoroughly investigated to print consistent membranes with uniform thickness. The membranes are fully characterized using SEM, FTIR, contact angle, and surface roughness measurements. The pure water permeability and separation performance of the 3D-printed membrane are further investigated and compared with those of commercial hydrophilic PTFE membranes. The 3D-printed membranes show similar permeability values to those of commercial membranes and could successfully separate oil droplets from oil-in-water emulsions. The membranes’ permeability is further predicted using a 1D tube model and numerical modeling. The effect of material’s property (e.g., swelling) and pore deformation during pressurization are studied to understand the discrepancy between the calculated and the experimental permeability values. The results provide valuable insights into the permeability prediction of 3D-printed membranes and the corresponding design optimization.

## Graphical abstract

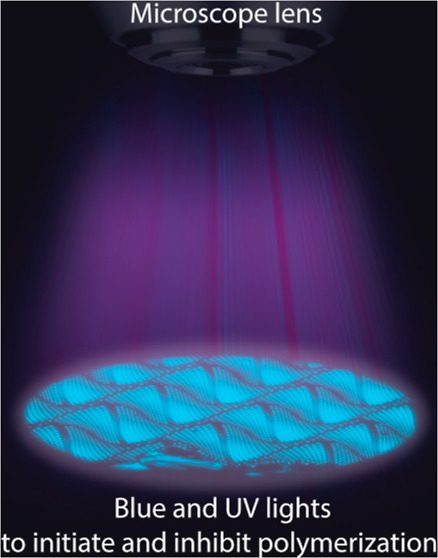

## Introduction

Membranes are selective barriers regulating the transport of substances between two compartments. They are used in industrial processes for separating various kinds of phases.^1,2^ Compared to conventional chemical separation processes, membrane separation techniques serve as green and sustainable alternatives. This is mainly due to low energy consumption, low pollution, and little use of additives and/or chemicals.^3^ On the contrary, the current membrane manufacturing process cannot be considered as a sustainable process as it heavily relies on hazardous solvents (e.g., *N*-methyl-2-pyrrolidone (NMP) or tetrahydrofuran (THF)) and nonbiodegradable polymers, causing harm to human health and the environment.^4^

Currently, in industrial and academic settings, the widely used method for membrane fabrication is the nonsolvent induced phase separation (NIPS).^5–7^ In this method, a homogeneous polymer solution (dope solution) containing a polymer and a solvent is prepared. This dope solution is spread out into a thin film with required thickness using a doctor blade. The film is subsequently submerged into a nonsolvent bath (coagulation bath), where the nonsolvent gradually mixes with the solvent, diffusing the solvent out of the polymer networks and leaving pores in its place.^8–10^ An alternative widely used method is the thermal induced phase separation process (TIPS). In this method, polymer and solvent are mixed at high temperature, making the dope solution. This solution is subsequently cooled or quenched, leading to the phase separation. A polymer-rich and a polymer-lean phase are formed, making, respectively, the membrane solid matrix and pores.^11^ Other membrane production methods include but are not limited to the following:

(i) Track etching, in which pores are formed in a polymer solid film via injecting multiple ion dose(s), making an ion track.^12,13^

(ii) Electrospinning, in which a high electric field is applied to the charged polymer solution, generating nanofibers.^14,15^

(iii) Sintering, in which a ceramic/polymer support is first produced. This support is coated using a coating solution containing ceramic or polymer particles, producing a ceramic or polymer membrane, respectively. The coating is further compressed and heated with the support, forming pores as the spaces between the compressed particles.^16^

The main problems of traditional membrane manufacturing methods are difficult optimization of the final product and low reproducibility from membrane to membrane. This is because conventional methods can only be manipulated via parameters such as bath temperature, composition of the bath, and duration of irradiation. If these parameters are not controlled properly, it can lead to poor quality and irregularities of the product, causing inconsistencies in the membrane performance.^17–19^

Recently, additive manufacturing, which is widely known as 3D printing, for membrane production has gained attention within academia and industry alike, with the number of publications rising from less than 20 in 2010 to more than 200 in 2019.^20^ By utilizing additive manufacturing, the shapes, sizes, designs, and the material of the desired membrane can be optimized in a much simpler way. It exhibits high consistency in the product quality and a narrow pore size distribution in the printed membrane. Using 3D printing, not only membranes but also spacers and entire membrane modules can be fabricated, which potentially lowers the production time and costs.^21–23^ These advantages make 3D printing an attractive alternative for membrane production.

Numerous 3D printing methods have been implemented so far for membrane production. Xing et al. manufactured a superhydrophobic membrane, inspired by lotus leaf, via the fused deposition modeling (FDM) method with poly­(lactic acid) (PLA) filaments.^24^ The membrane was further treated using chemical etching to reduce the pore size and change the surface roughness. The treated membrane had a pore size of 40–600 μm which exhibited a good oil–water separation performance from a mixture of oil–water with a 1:1 ratio. Yuan et al. utilized the selective laser sintering (SLS) method with polyamide powder to produce a single thin layer.^25^ They demonstrated that the membrane characteristics, e.g., porosity, wettability, and morphology, could be optimized by manipulating the parameters of the laser, namely, power, the distance between each laser track (hatch space), and the scanning count. Jin et al. used digital light processing to print ceramic membranes from Al_2_O_3_ slurry in a layer-by-layer manner.^26^ The printed structures were sintered by subsequent exposure to high temperature to evaporate the solvent and bind the metal. To further enhance the oleophilic and hydrophobic properties, the ceramic membrane was dip-coated into a silicon dioxide (SiO_2_) solution forming a layer of SiO_2_ nanoparticles on the membrane surface. Superhydrophobic membranes with a pore size of 300–700 μm and a water contact angle of 162° were fabricated. The membranes were also mechanically stable and could withstand harsh chemicals. By utilizing a digital light processing (DLP) 3D printer, Femmer et al. produced a three-dimensional polydimethylsiloxane (PDMS) membrane with effective gas transfer properties.^27^ They printed a cross-flow gas–liquid contactor using a PDMS membrane (1 mm thick) directly attached to the exchange channels. The module was tested with O_2_, CO_2_, and N_2_ to determine the pure gas permeability. Compared to standard PDMS membranes, the printed ones exhibited lower permeability (ca. 15%) but similar selectivity.

Most commercial 3D printing systems have a resolution of ca. 50–100 μm, which further limits the smallest printable pore size in a membrane.^28^ Due to this resolution limit, microfiltration (MF) membranes with a sub-10 μm (0.1–10 μm) pore diameter cannot be directly 3D-printed. MF membranes are an important category of membrane filtration with a wide range of applications, ranging from wastewater treatment^29,30^ to food processing (e.g., bacteria removal).^31^ One strategy to print MF membranes using current 3D printing techniques is post-treating the 3D-printed membrane. Methodologies such as polymerization-induced phase separation (PIPS) can be implemented to make pores smaller than the resolution limit.^32^ Using this method, however, the precise control of the pore size and shape is still a challenge.

Currently, only photopolymerization has the potential to print MF membranes.^33^ In the photopolymerization process, photoreactive polymers or photopolymers are cured using a laser, ultraviolet (UV) or visible light with a resolution limit of around 15 μm.^34,35^ Using two-photon polymerization (TPP), the resolution limit can go as low as 0.15 μm. This difference in resolution limit can be explained by the various polymerization areas in a light cone. In a typical photopolymerization process, photopolymers are made by mixing the monomers with lightweight photoinitiators.^36,37^ Polymerization is initiated by exposure of the photopolymers to the UV light, leading to the final product. In the TPP method, two photons, instead of one, are utilized to induce polymerization, resulting in a higher resolution.^38,39^ Due to the relatively small polymerization point, the TPP process is an extremely slow process, making it inappropriate for printing larger areas. Because the resolution limit of the standard photopolymerization method is around 15 μm, and the printing speed of the TPP process is slow, these two techniques are not suitable for printing MF membranes.

Recently, a new 3D printing method, namely, dual-wavelength stereolithography (DWS), has been developed. First developed by De Beer et al.,^40^ the 3D printer utilizes an initiation and an inhibition wavelength to initiate and inhibit photopolymerization, respectively. By adding a photoinhibitor to the photopolymer, a termination process of polymerization can be achieved upon reaction of the photoinhibitor with the inhibition wavelength. This limits the polymerization boundaries without sacrificing the writing speed. Comparing to the standard photopolymerization method and the TPP printers, this system has the advantage of high resolution and fast printing.^38,39^ The recent advances in this field have led to the development of the so-called dual-wavelength volumetric microlithography (DWVML).^41^ In contrast to the sequential printing methods (e.g., two-photon lithography (TPL)), the printing speed in DWVML is not determined by the in-fill factor of the printed structure. This characteristic makes DWVML an ideal choice for printing structures with high in-fill and aspect ratios, such as membranes, meshes, and molds.^41^ The difference in the printing process between two-photon polymerization and the DWVML is schematically illustrated in Figure 1.

**Figure 1:**
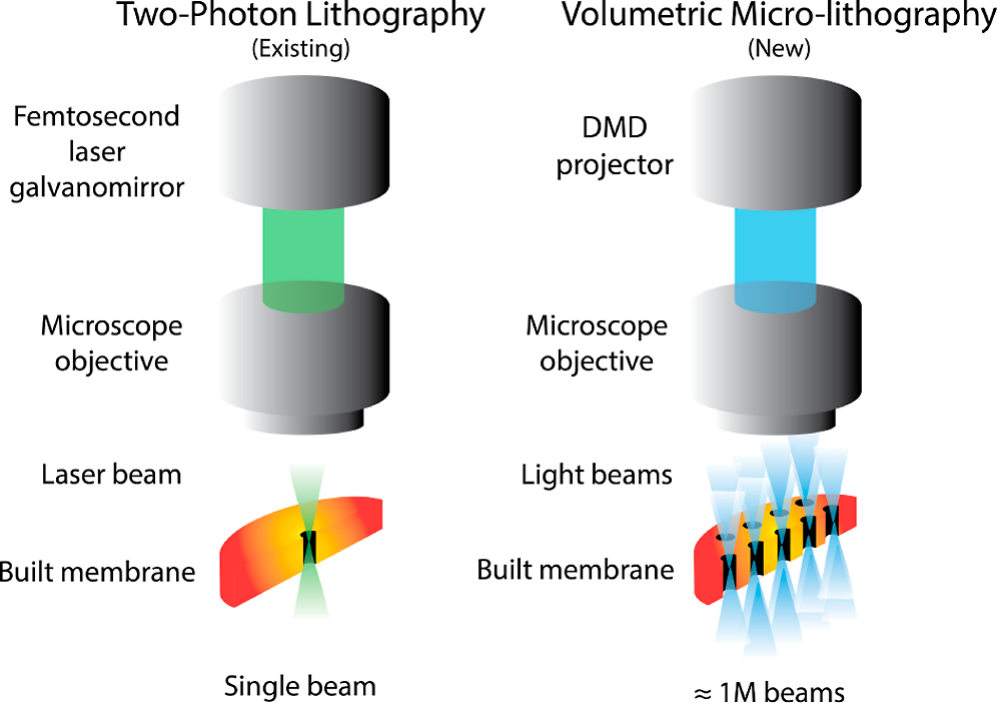
Comparison between the existing two-photon lithography (TPL) and the novel dual-wavelength volumetric microlithography (DWVML) for 3D printing porous membranes. In DWVML, the nano- or microsized pores are constructed using million light beams (not a single beam), providing a faster and cheaper 3D printing technique compared to the TPL method (DMD is a digital micromirror device). The relatively small polymerization point in the TPL method makes it a slow process, which is not suitable for printing large areas.

In this study, we directly 3D print MF membranes with a pore diameter of ≈10 μm using a dual-wavelength microstereolithography printer with a resolution of 1.368 μm.^42^ The resulting membranes are characterized using scanning electron microscopy (SEM), atomic force microscopy (AFM), Fourier transform infrared spectroscopy (FTIR), and contact angle measurements. The pure water permeability and separation performance of the membranes are further evaluated using monodisperse oil-in-water emulsions. These results are compared with those obtained from a commercial hydrophilic membrane with the same surface pore size (PTFE omnipore, JCWP14225, Merck, Germany) to evaluate the performance of the 3D-printed membranes. Membranes’ permeability values are predicted using a 1D tube model based on membrane porosity and pore radius. Numerical simulations are further performed, using the open-source software OpenFOAM, to better understand the discrepancy between the theoretical and experimental permeability values. It provides insight into the effect of materials’ properties and pore deformation on predicting membranes’ permeability, paving the way for 3D-printed membrane design optimization.

## Experimental Section

### Materials

All the chemicals are purchased from Sigma-Aldrich, The Netherlands, unless otherwise specified. Photoinitiator, co-initiator, and photoinhibitor are, respectively, camphorquinone (CQ), ethyl 4-(dimethylamino)­benzoate (EDAB), and 2,2′-bis (2-chlorophenyl)-4,4′,5,5′-tetraphenyl-1,2′-biimidazole (o-Cl-HABI). Poly­(ethylene glycol) diacrylate with a molecular weight of 700 g/mol (PEGDA 700) is used as the monomer. Ethanol (VWR, analytical grade >99.8%) is used as the cleaning solvent. Isopropanol (IPA) (VWR, analytical grade >99.8%) and methyl isobutyl ketone (MIBK) (analytical grade >99.0%) are used as the solvent in the postprinting step to remove residual resin. For making oil-in-water emulsions, *n*-hexadecane (reagent plus 99%) is used as the oil phase, oil red EGN as the oil-soluble dye, and sodium dodecyl sulfate (ACS reagent, ≥99.0%) as the aqueous-based surfactant. The commercial hydrophilic polytetrafluoroethylene (PTFE) membrane (Omnipore, JCWP14225) is purchased from Merck, Germany.

### Printing System

The printing system is schematically shown in Figure 2. The microscope lens, which collects the light and projects it into the resin, is used to determine the *x*–*y* resolution of the printing. Since the microscope operates in its reverse order, the image which is projected on the resin is minimized into a smaller projection image. This makes the basis of the printing process giving the printing resolution of

1 $$R_{xy}=14/M$$

in which *R*_*xy*_ is the resolution in the *x*–*y* plane (μm) and M is the microscope lens magnification. In this work, a microscope magnification of 10*x* is chosen giving a resolution of 1.4 μm.

**Figure 2:**
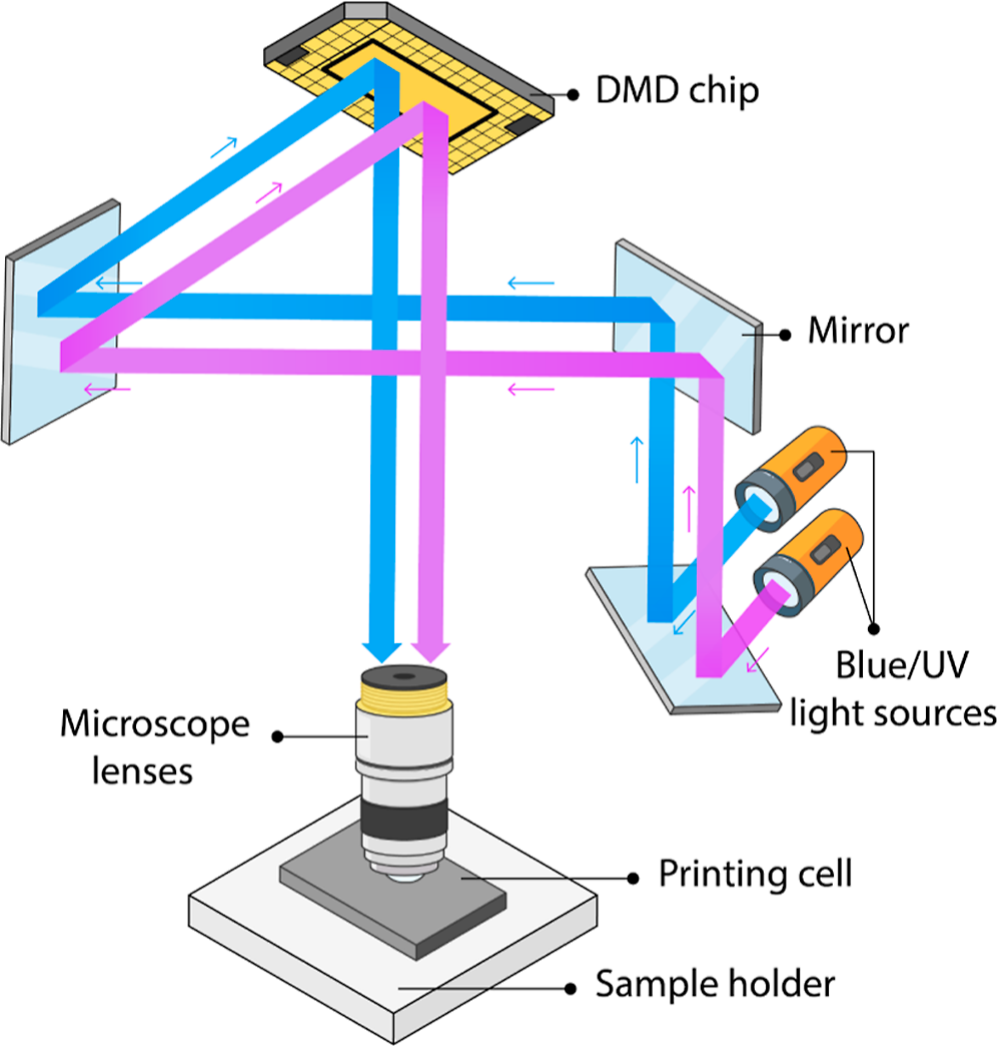
Schematic illustration showing the simplified 3D printer and the printing process. The microscope lens collects light and projects it into the resin. The lens magnification determines the *x*–*y* resolution of the print (see eq 1). Blue light (λ = 460 nm) and UV light (λ = 380 nm) are produced from a digital light processing (DLP) unit to initiate and inhibit polymerization, respectively. The blue and UV lights are projected in a layer-by-layer fashion in millisecond sequences to print the membrane tile (see Figure 4a). During tile printing, the microscope lenses move downward. They get back to their original position after printing. The next tile is printed via movement of the sample holder.

The printing system utilizes a digital light processing (DLP) unit to produce blue light (λ_blue_ = 460 nm) and UV light (λ_UV_ = 380 nm) as the initiation and inhibition wavelengths, respectively. The photoinitiator (CQ) and co-initiator (EDAB) are initiated using the projected area with blue light (Figure 3), leading to the polymerization of the PEGDA monomer. Areas projected with the UV light initiate the photoinhibitor (o-Cl-HABI), inhibiting polymerization.^43^ Via tuning the projection area and intensities of the light sources, structures such as membranes can be printed.

**Figure 3:**
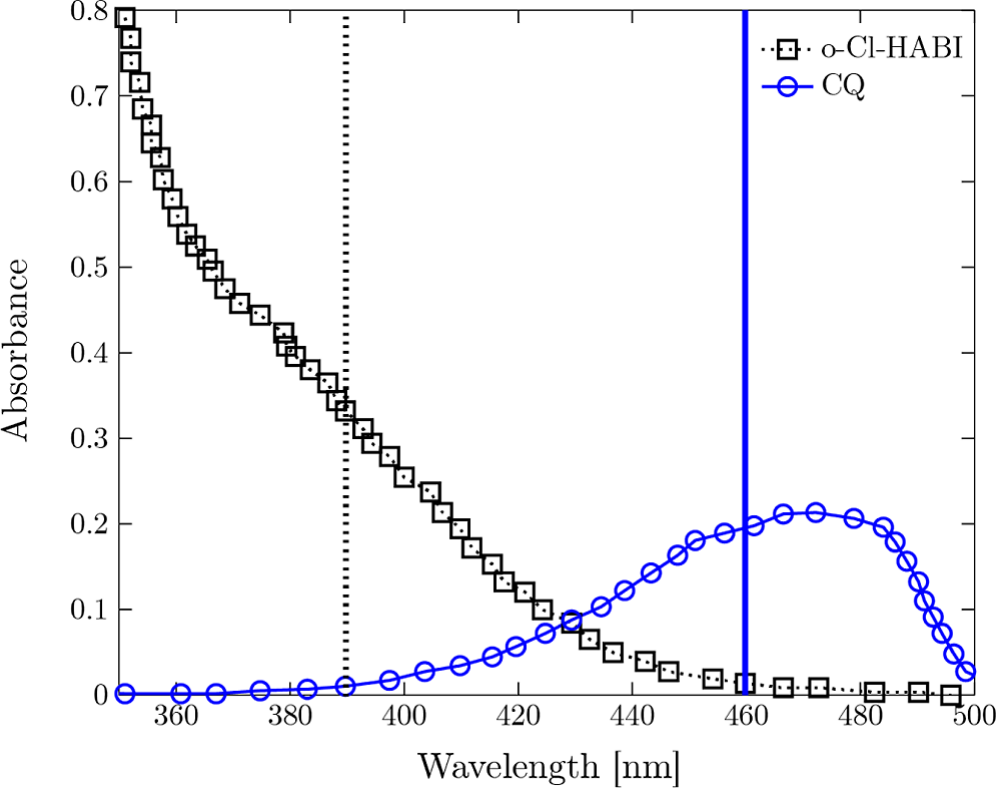
Absorbance of the photoinitiator (CQ) and the photoinhibitor (o-Cl-HABI) providing information on the suitable wavelengths for initiation of these chemicals, respectively (the vertical black dotted and blue solid lines refer to the wavelength range of the UV and blue lights, respectively).

In a layer-by-layer fashion, the UV and blue lights are projected in short sequences (millisecond range). The downward movement of lenses makes the printing process similar to printing a bulk volume. In other words, instead of printing a thin layer, a volume is printed in a very short time. Thus, the target is printed in a volume-by-volume fashion, rather than a layer-by-layer fashion. Further details of the printing process, calculation of the light intensities, and rate of polymerization are provided in the Supporting Information, section “Membrane design calculation and printing process”.

### Membrane Design

Printing is performed in a tile-by-tile fashion. After each individual tile is designed, the printer prints every tile until the final number of tiles is reached. For printing a membrane, the number of pores, pore diameter, pore arrangements, and thickness of a tile are designed beforehand. These respective parameters in this study are 256 pores (16 pores in *x* and 16 pores in *y* directions), 10 μm pore diameter, and 70 μm membrane thickness, making the target model for membrane printing.^42^ Figure 4 shows the top and side view of the designed target model (tile) for the membrane. The desired conversion rate at every *x*, *y*, and *z* pixel value of the membrane is further depicted. The gradient descent method is used to calculate the light projections via an iterative procedure. The calculation of the conversion and light projections are described in the Supporting Information, section “Membrane design calculation and printing process” (eqs S4–S6). The final calculated light projection area and intensities (printing model) are used as the input into the 3D printer. The light intensities and chemical conversion at each location along with a flowchart depicting the membrane design calculation procedure are further shown in the SI, Figures S1–S3. For additional details, the reader is referred to the work by Mulder et al.^41^

**Figure 4:**
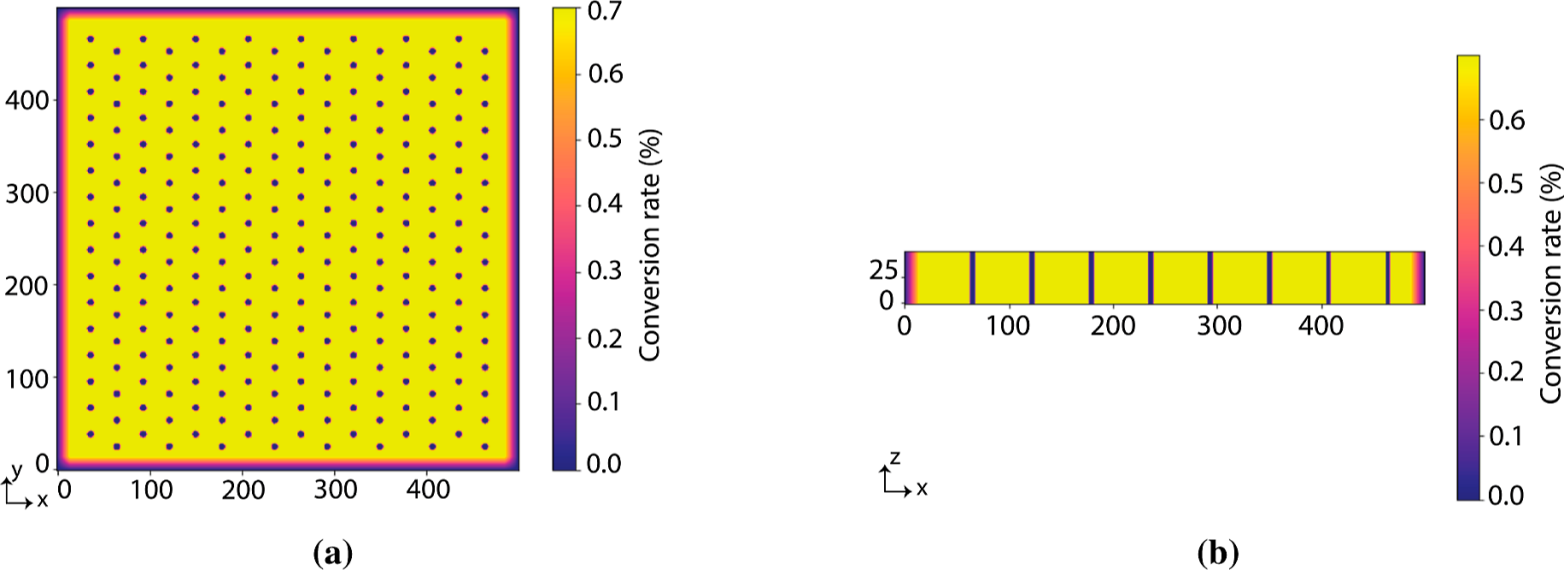
(a) Top view and (b) side view of the desired membrane target model. The *x*, *y*, and *z* values are pixel numbers and the color bar shows the conversion rate in percentage. Via a mixture of blue light and UV light projection, in which the pore area has a high intensity of UV light and the solid area has a high intensity of blue light, a membrane structure can be printed.

### Membrane Printing Process

The photopolymer resin is produced via mixing the photoinitiator (CQ), co-initiator (EDAB), and the photoinhibitor (o-Cl-HABI) with PEGDA monomer. The resin is used to fill the printing cell, which is fabricated using the following procedure: (1) Micro cover glasses (24 × 24 mm^2^, Brand, Germany) and microscope glasses (76 × 26 mm^2^, Brand, Germany) are cleaned with ethanol and dried using a lab-sized high-pressure blower. (2) Spacer beads of 70 μm (Micropearl SP series, Sekisui Chemical Co., Ltd., Japan) are mixed with a small amount of the photopolymer resin with a concentration of 0.0033 g/mL. Ten microliters of the mixed resin and beads are then dotted on the four corners of the micro cover glass. (3) A cover glass is placed on top of the four dots, made from the mixed resin and beads, to form the printing cell (see SI Figure S4). Using a pipet, 50 μL of the resin is then introduced from the side to fill the cell and the space between the top and bottom glasses. This cell design and the filling procedure along with the order of tile printing (see Figure 6) eliminate the possibility of membrane thickness variation, leading to reproducible thickness values of 70 μm. The thickness variation can possibly be caused by a not fully filled printing cell by the resin or collapse of the top cover glass.

The printing model, described in the section Membrane Design, is then read by the printer to start the printing process which is schematically shown in Figure 5. The membrane is printed using both layer-by-layer and tile-by-tile fashions. Over a time span of 5 s, each tile is printed layer-by-layer. To achieve a membrane size of 1 × 1 cm^2^, 256 tiles are printed, consisting of 16 tiles in the *x* and 16 tiles in the *y* direction. The polymerized resin on the edge of each tile guarantees automatic stitching of the tiles. The printing process starts from the focus point of the lens, which is the bottom of the filled printing cell with the resin (top of the bottom glass). When printing starts, the lenses project light in a blue-UV-blue-UV sequence, while moving upward. Once printing one tile is finished, the lens gets back to its original position, namely, its focus point (top of the bottom glass). The next tile will be printed via movement of the sample stage to the next position. When the desired sample size (here a 1 × 1 cm^2^ membrane) is reached, the printing stops.

**Figure 5:**
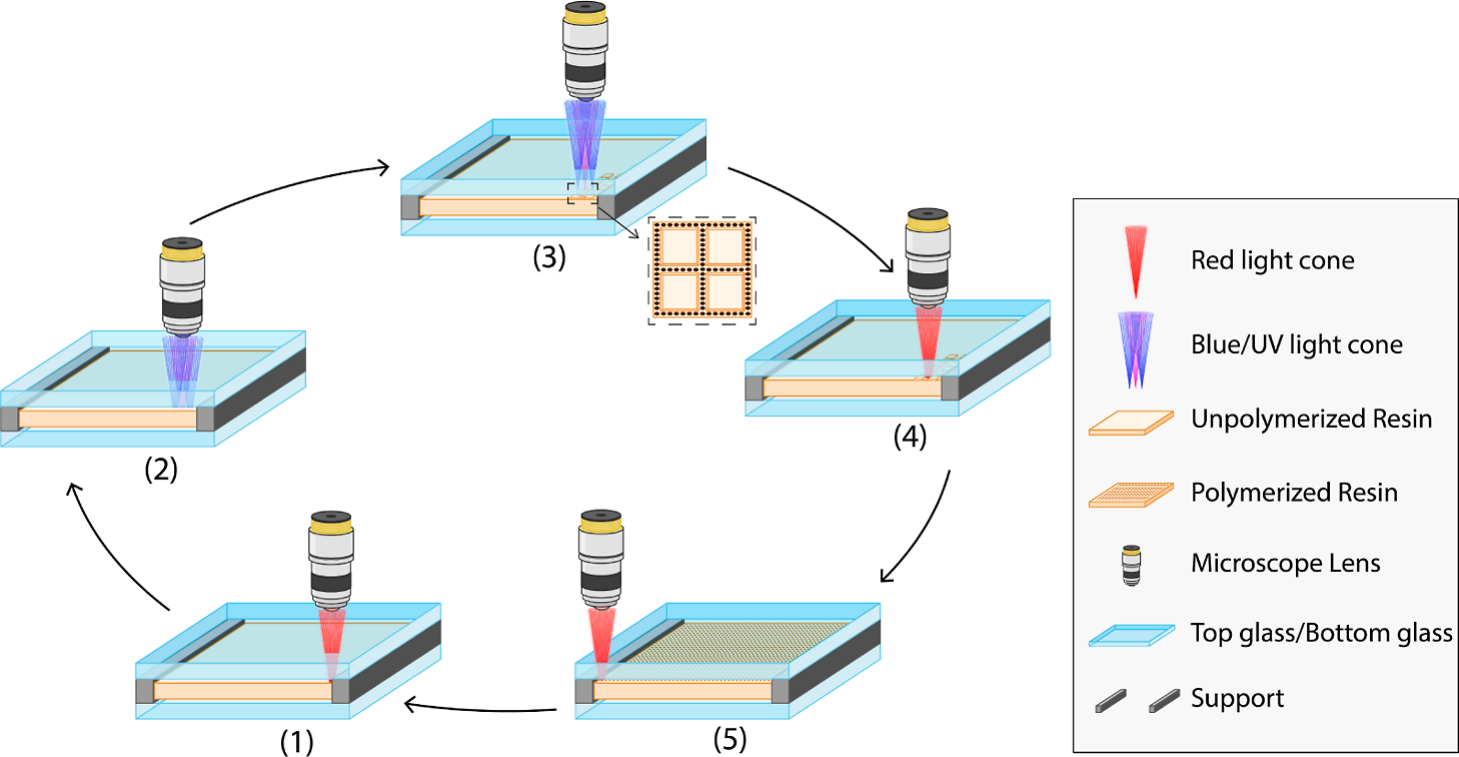
Illustration of the membrane printing in a tile-by-tile fashion. (1) The lens is focused on the bottom of the printing cell (top of the bottom glass) using red light. (2) While the lens moves upward, the UV/blue light starts flashing. (3) A small volume of the resin is cured. (4) The lens gets back to its original focus point on top of the bottom glass. (5) The lens moves to the location of the next designated tile. This process is continued until the desired structure is printed. During this process, the printing area is visualized using a camera illuminated by red light. The red light wavelength does not affect the resin.

The order of tile printing is an important parameter affecting the uniformity along the membrane thickness (see schematic illustration in Figure 6). Printing the tiles along one direction leads to the collapse of the top glass, resulting in an uneven membrane thickness, which is thinner on one side compared to the opposite side (Figure 6a). To prevent this effect and nonuniformity in membrane thickness, first support tiles are printed (four in the corners and one in the middle of the membrane). The full membrane is then printed in sequence (Figure 6b).

**Figure 6:**
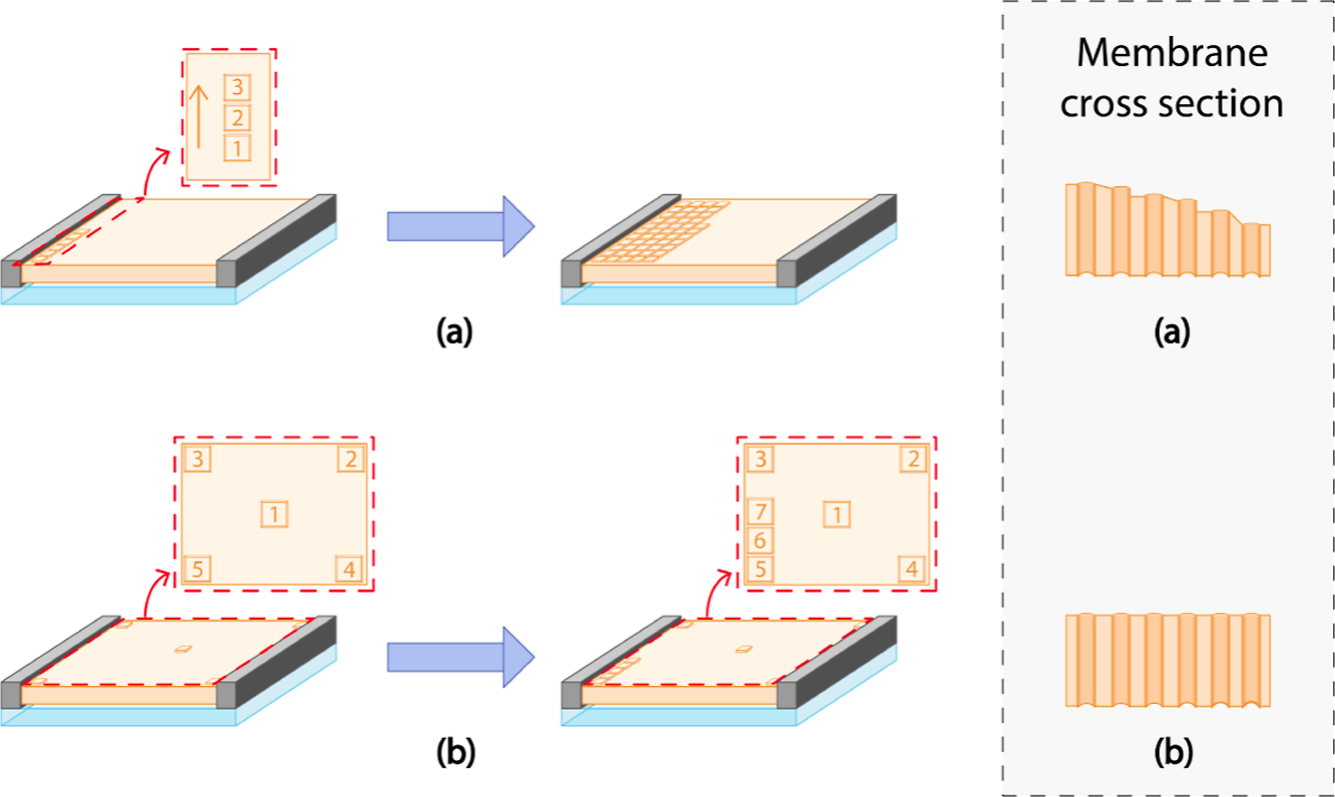
Illustration of the effect of the tile printing order on the uniformity of the membrane thickness. (a) Printing along one side (from one end to another) leads to nonuniform membrane thickness due to the collapse of the top glass on one side. (b) Printing support tiles as four in the corners and one in the middle guarantees a membrane with uniform thickness.

As the final step, the printed membrane is taken out and washed using a mixture of isopropanol and methyl isobutyl ketone (MIBK). This washing step guarantees the removal of the unpolymerized resin,^44^ which would be inside the membrane pores. The postprocessed membrane is heated using a hot plate (IKA C-MAG HS-7) at a temperature of 160 °C for 20 min to increase the cross-linking density. This is an important step to further improve the mechanical strength of the printed membrane.^45^ To prevent local swelling and the subsequent cracks in the polymer material,^46^ the membrane is kept in water for 1 h before further testing.

### Membrane Characterization

#### Microscopy Imaging

Scanning electron microscopy (SEM) (JSM-6010LA, JEOL, Japan) with an acceleration voltage of 10 kV is used to observe the morphology of the top, bottom, and cross section of the 3D-printed and commercial membranes. The cross-section samples are prepared by cutting the sample using liquid nitrogen. All samples are coated with gold using a sputter coater (JFC-1300, JEOL, Japan) with a current of 20 mA for a duration of 30 s.

Atomic force microscopy (AFM) (Dimension Edge, Bruker, USA) is used to investigate the roughness of the printed and commercial membranes. The root-mean-square roughness of the membranes is measured in tapping mode.

#### Pore Size Distribution

The pore size distribution of the 3D-printed membrane is performed via image processing of SEM images using FIJI ImageJ.^47^ The SEM image is imported into ImageJ and is further enhanced by adjusting the threshold. To check reproducibility, two membranes are processed via ImageJ, and the final pore size distribution is obtained by taking the average of the two distributions. The adjusted threshold SEM image along with the details on the process of the threshold adjustment and pore size measurement are shown in SI Figure S5 and section “Pore size distribution measurement: 3D-printed membrane”.

The pore size distribution of the commercial PTFE membrane (JCWP14225) is measured using a capillary flow porometer (POROLUX Revo). In this measurement, the membrane is first infused with a low surface tension wetting liquid (Porefil, wetting liquid for porometers, fluorinated hydrocarbon, Porometer, Belgium). Nitrogen gas is then pushed through the membrane at different pressure values leading to the displacement of the wetting liquid. The corresponding gas flow rate is measured simultaneously (see Supporting Information section “Pore size distribution measurement: commercial PTFE membrane” for more details on the experimental procedure and the flow vs pressure plot (Figure S6)).

#### Contact Angle Measurements

An optical contact angle goniometer (OCA 25, DataPhysics) is used to measure the contact angles of air and *n*-hexadecane on membranes immersed in water, as well as that of water and *n*-hexadecane on membranes in air. Both static and dynamic contact angle measurements are performed as detailed below. The static contact angle measurements are performed using a 6 μL droplet. The sessile drop method using water and *n*-hexadecane as the test liquids is used to perform the water-in-air and oil-in-air experiments, respectively. The captive bubble method using a 6 μL droplet of either air or *n*-hexadecane is used to conduct the underwater experiments, i.e., air-in-water and oil-in-water, respectively. Dynamic contact angle measurements are only performed for water-in-air on both 3D-printed and commercial membranes. In this measurement, a 2 μL water droplet is first dispensed on the membrane, in which the needle is submerged. Then an advancing and receding contact angle (ARCA) setting is then set, in which the droplet volume is increased to 6 μL at the rate of 0.5 μL/s for 6 cycles. The advancing and receding contact angles are measured within 6 s each, with a 2 s pause in between. The contact angle values are detected and recorded automatically by the OCA software.

#### Pure Water Permeability Measurements

The permeability of both 3D-printed and commercial membranes is measured using a custom-built dead-end filtration setup (Figure 7). A membrane module accommodating a 1 × 1 cm^2^ membrane has been designed and fabricated (considering the width of the sealing O-ring, the effective membrane area is 5.5 × 5.5 mm^2^). The membrane is placed in the membrane module and degassing is performed by circulating the pure water (Milli-Q grade) across the membrane without pressurizing. This is achieved by closing the permeate outlet and opening the degassing port. After the degassing step, pressure is increased stepwise from 100 to 500 mbar with increments of 100 mbar using an OB1 pressure controller (OB1Mk3+, ElveFlow, France). The permeating flow rate is measured simultaneously using a flow meter (Bronkhorst Coriolis flow meter).

**Figure 7:**
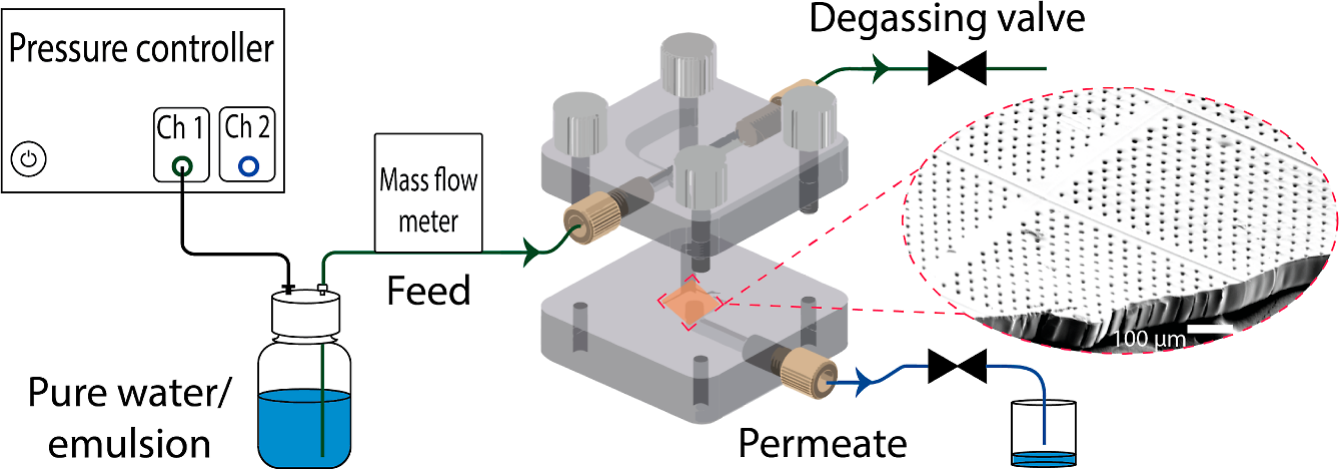
Schematic illustration of the dead-end filtration setup utilized for pure water permeability and separation experiments using a custom-built membrane module, along with the SEM image of the 3D-printed membrane (scale bar is 100 μm).

### Separation Experiments

#### Sample Preparation and Rejection Ratio Calculations

Separation performance of both 3D-printed and commercial membranes is investigated via filtration of monodisperse oil-in-water (O/W) emulsions using the dead-end filtration setup (Figure 7). The system is set at 500 mbar for 5 min and the corresponding permeate is collected. Images of the feed and permeate are taken using a digital microscope (VHX-7000 digital microscope (KEYENCE, USA)). Image processing using FIJI ImageJ^47^ is further implemented to obtain the concentration of the oil droplets. This concentration is first measured for the concentrated O/W emulsion directly obtained from the droplet generator (see section Fabrication of Monodisperse O/W Emulsion). For reproducibility purposes, in each measurement, five feed/permeate samples for each SDS concentration are analyzed. The diameter of individual droplets is measured in FIJI ImageJ, at least ten times to get the average droplet diameter and the corresponding volume. The calculation procedure to obtain the average concentration of each emulsion is also performed three times. The details of the calculation of oil concentration along with the microscopy images are shown in the Supporting Information section “O/W emulsion preparation: Concentrated feed” and Figure S7.

The concentrated O/W emulsion is further diluted using corresponding aqueous surfactant solution to prepare the feed samples for separation experiments. The concentration of the oil droplets in the feed (*C*_f_) and permeate (*C*_p_) is measured similarly based on droplet size measurements. For consistency purposes, 0.2 μL of the feed or permeate is placed on a microscopy glass slide to take the images. The dilution procedure and images of the diluted feed emulsions are shown in the Supporting Information, section “O/W emulsion preparation: Diluted feed” and Figure S8. The corresponding concentration values are subsequently calculated to obtain the rejection ratio (*R*) using

2 $$R=\left(1-\left(\frac{C_{p}}{C_{f}}\right)\right)\times 100$$

#### Fabrication of Monodisperse O/W Emulsion

Monodisperse O/W emulsion is fabricated using a focused-flow droplet generator microfluidic chip (Micronit Microfluidics B.V., The Netherlands).^48^ The schematic of the setup is shown in Figure 8a. The chip consists of a cross-junction configuration with a mid channel and two side channels (see Figure 8b). The dispersed phase (oil) is pumped through the mid channel, while the continuous phase (aqueous-based surfactant solution) squeezes into the oil phase through the side channels, generating monodisperse oil droplets. A pressure controller (OB1Mk3+, ElveFlow, France) with two channels, namely, high-pressure channel 3 with a maximum of 8000 mbar and low-pressure channel 4 with a maximum of 2000 mbar, is used to pressurize water and oil into the corresponding channels. This pressure controller is connected to the nitrogen gas line at 7000 mbar. The bottle of sodium dodecyl sulfate (SDS) surfactant solution is connected to channel 4 and pressurized at 360 mbar. The oil bottle is connected to channel 3 and pressurized at 420 mbar. Two surfactant solutions have been prepared by dissolving SDS at concentrations of 50% and 100% of the critical micelle concentration (CMC) in pure water (Milli-Q grade). These surfactant solutions are referred to as SDS50 and SDS100, respectively, with the numerical value indicating the SDS concentration as the percentage of the CMC. The CMC of SDS was experimentally determined to be 8.1 mM within a temperature range of 20–25 °C.^49^ Sufficient time, exceeding 12 h, has been allotted to completely dissolve the surfactant in water. The oil phase (*n*-hexadecane) has been dyed using 0.04 g of oil-red EGN and filtered using a Whatman filter unit (0.45 μm) before O/W emulsion fabrication. The images of the fabricated O/W emulsions (concentrated and diluted) are shown in SI Figures S7 and S8, respectively.

**Figure 8:**
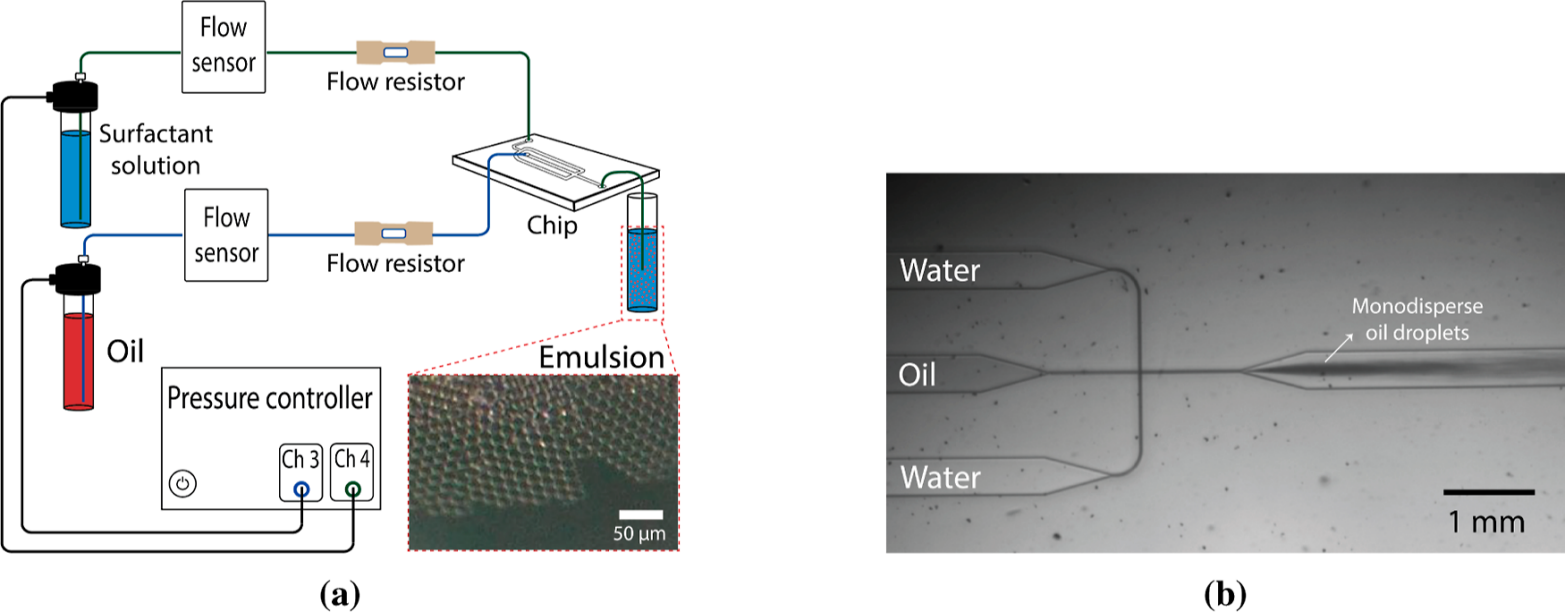
(a) Schematic illustration of the microfluidic emulsion fabrication setup to prepare monodisperse emulsions using a commercial microfluidic chip (focused-flow droplet generator), along with the microscopy image of the fabricated emulsion (scale bar is 50 μm)). (b) Optical microscopy image of the chip used for emulsion fabrication.

### Water Uptake Ratio Calculations

Prior to the permeability and separation experiments, both 3D-printed and commercial PTFE membranes were kept in water (Milli-Q grade) for 1 h. The water uptake ratio (WUR) of both membranes was calculated using

3 $$WUR=\frac{t_{wet}-t_{dry}}{t_{dry}}\times 100$$

where *t*_wet_ and *t*_dry_ are thickness of the wet and dry membranes, respectively. The wet thickness was measured using a digital micrometer (Mitutoyo, MDC-25PX), while the dry thickness was obtained via image analysis of the SEM cross-sectional images using FIJI ImageJ.^47^

### FTIR Spectroscopy Measurements

FTIR measurements on 3D-printed membranes are performed using a Nicolet iS50 FTIR spectrometer (Thermo Fisher Scientific). Fresh samples (dry) as well as those that have already been kept in water in closed glass containers in the lab environment (T ≈20 °C) for different periods of time, namely, 2 weeks and 3 months, are tested to investigate the possible hydrolysis and degradation of PEGDA material. The dry fresh sample is first cleaned with a nitrogen gas stream to remove any dust. The samples that have been kept in water are first rinsed with IPA and water to remove any organic and/or inorganic residues. They are further dried in a vacuum oven at 40 °C to remove all the water and solvent residues followed by cleaning with the nitrogen gas stream. For each condition (dry and wet membranes), three samples (a small piece of ≈10 mg) are tested. Since similar IR spectra are observed for each condition, only one spectrum is further analyzed (see Supporting Information, section “FTIR results”).

## Computer Simulations

### Flow Simulations

3D-printed membranes with well-defined cylindrical pore shape offer the possibility of membrane pure water permeability prediction. This is currently not straightforward in a bulk porous medium, e.g., commercial polymeric membranes, due to undefined pore shape and pore tortuosity.^50^

Here, numerical simulations are used to replicate experiments and predict the pure water permeability of the 3D-printed membranes. The simulations are performed in the open-source software package OpenFOAM,^51^ using simpleFoam, a steady-state solver for incompressible flow based on the SIMPLE (Semi-Implicit Method for Pressure-Linked Equations) algorithm^52^ on a workstation with an Intel Xeon Silver 4214R CPU.

Compared to the experimental design (see section Membrane Design), the simulation is restricted to a reduced unit cell with 5% porosity, containing 16 pores with hexagonal distribution and a diameter of 10 μm due to computational limitations and the periodicity of the membrane. An illustration of the unit cell geometry, which is periodic on all sides, is shown in Figure 9a. In the simulation, only the fluid domain is modeled, and the interaction between water and the PEGDA material of the membrane is disregarded. Modeling of the fluid domain is performed not only within the membrane region but also in the regions before and after the membrane’s inlet and outlet, respectively. The fluid entry and exit regions are set to be each 50 μm long (Figure 9b). The entire domain is discretized into a mesh with an average element size of 1 μm.

**Figure 9:**
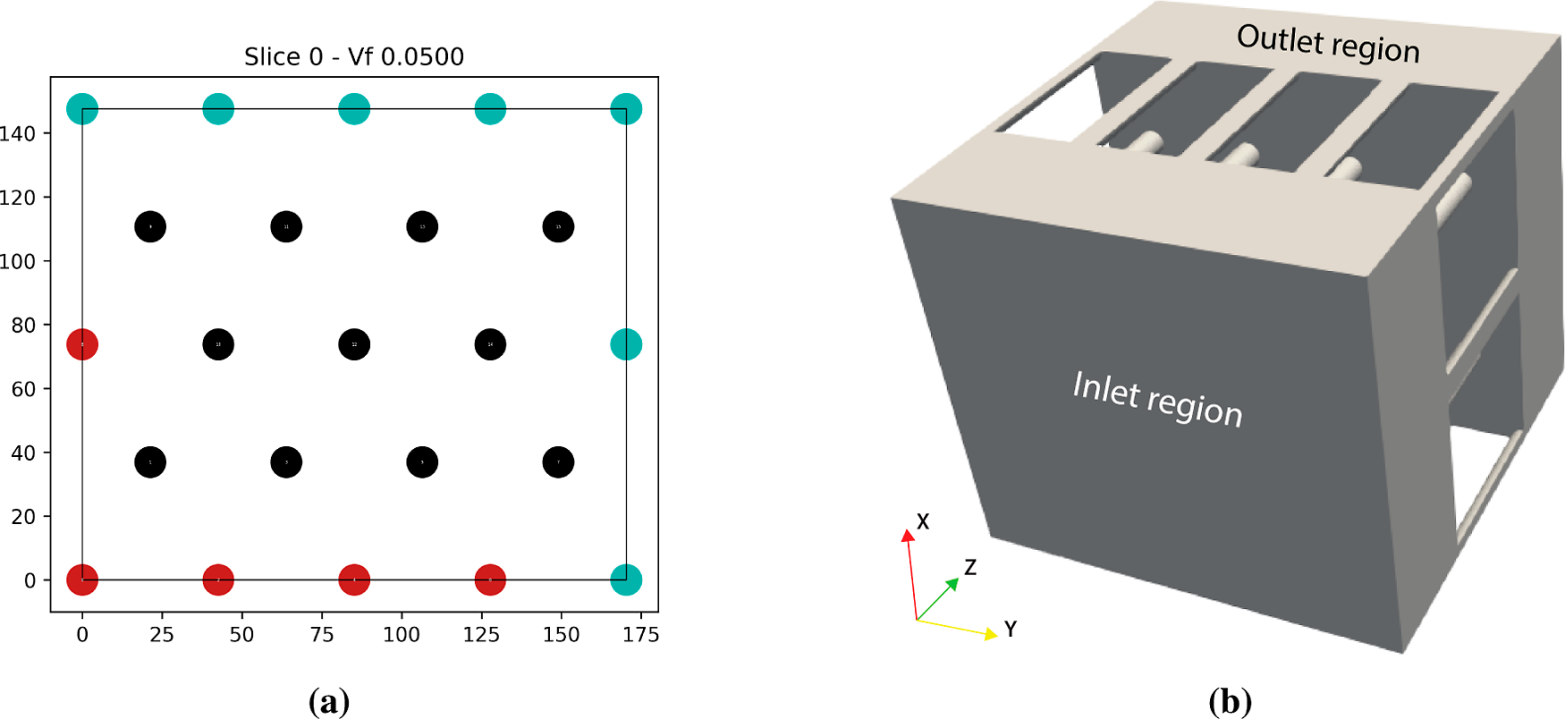
(a) 2D view cut of the geometry used to model the 3D-printed membrane. The view cut is perpendicular to the flow direction, i.e., coplanar with the membrane. The black circles show pores inside the unit cell, while the red circles show pores on its boundary. The teal circles refer to the periodic counterparts of the red circles. (b) The fluid numerical domain, consisting of the 3D pores in addition to an inlet region and an outlet region used to enforce far field boundary conditions. Note that the fluid domain is the complement of the membrane geometry.

The physical properties of water, e.g., density and dynamic viscosity, are considered as ρ = 1000 kg/m^3^ and μ = 1 mPa.s, respectively. As the boundary conditions, a pressure of 1 kPa and 0 Pa was applied at the inlet and outlet, respectively, resulting in a drop of 1 kPa across the fluid domain. It is worth noting that the flow is laminar and the predicted permeability is independent of the pressure drop. Periodicity is enforced on the lateral sides of the fluid domain, and a no-slip boundary condition is assigned at interfaces between the fluid and membrane pores. The velocity profile within a circular pore is analyzed to validate the simulation. As expected for a Poiseuille flow,^53^ it exhibits a parabolic nature, with minimum values at the wall, where the no-slip condition is enforced, and maximum values along the center lines.

### Pore Deformation

To study pore deformation/compression during pressurization, Abaqus 2022 on a DelftBlue high-performance computer (HPC) is used.^70^ Compression simulation is performed on a quarter of a unit cell comprising one pore (Figure 10), where the fluid pressure in the pore (*P*_p_) decreases linearly from a maximum of *P*_c_ = 50 kPa in the inlet to 0 kPa in the outlet. This pressure profile is coherent with the expected pressure drop profile within the membrane region (see section Pure Water Permeability Measurements, section Simulation Results, and Figure 16b). The hydrogel membrane material is restrained from translating in the *z* direction at the outlet, while becoming free at the inlet, allowing for compression when pressure is applied.

**Figure 10:**
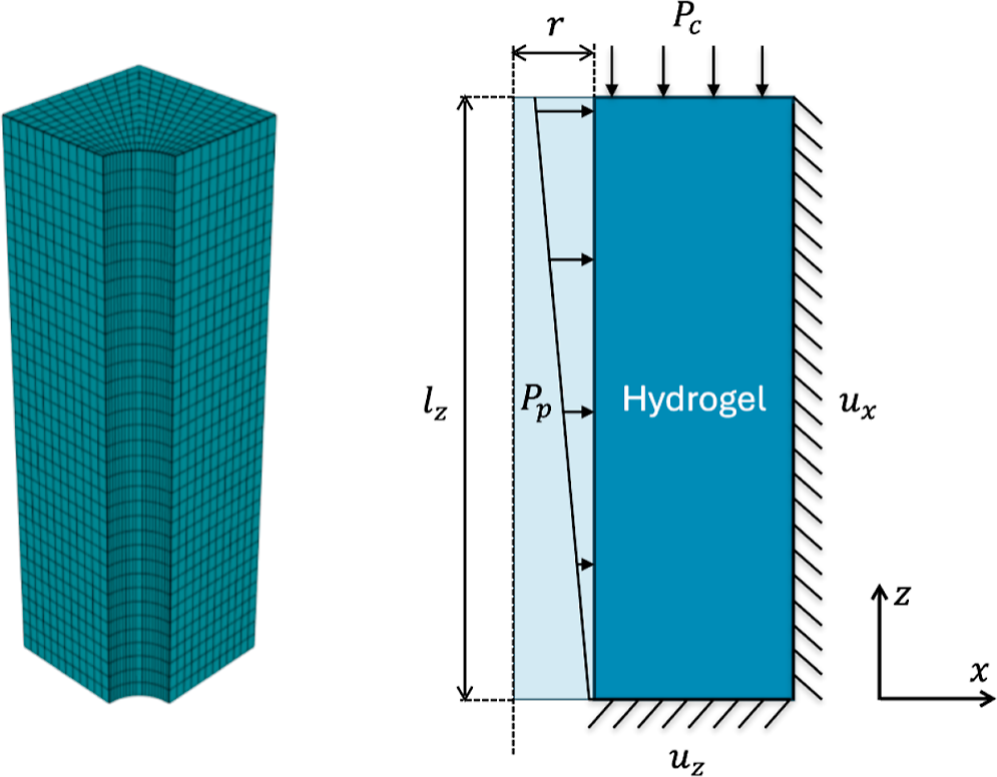
Setup for simulating a single pore surrounded by a hydrogel in Abaqus 2022. *l*_*z*_ denotes the membrane length (i.e., 70 μm), *r* is the radius of the pore (i.e., 5 μm), *P*_p_ is the fluid pressure in the pore, and *P*_c_ is the maximum pressure in the inlet (50 kPa).

Two cases are studied: (a) the hydrogel membrane is constrained and cannot expand in the *x* direction, and (b) the membrane is free to expand. The remaining boundary conditions are symmetric. The geometry is meshed with C3D20R elements. Following a mesh convergence study, the corresponding size is set to approximately 2 μm in the outer region, while it is smaller close to the pore.

The swelling is introduced through a dilatation coefficient and a temperature field to achieve the required amount of swelling. An implicit integration scheme (Abaqus Standard) is used. As the filter undergoes deformation, the pore length changes, and the pressure profile should be updated accordingly. This is not straightforward without implementing a user subroutine. Here, the model is solved iteratively, where the converged pore length is used to define the pressure profile of the next iteration until convergence is achieved.

The physical properties of the PEGDA material are taken from the literature as follows: density ρ = 1120 kg/m^3^ ^54^ and Young’s modulus of 118 kPa.^55^ Compression simulations are performed using different Poisson’s ratios (ν = 0.25, 0.3, 0.35, 0.4, and 0.45)^56^ and based on various volumetric swelling ratios (0, 0.1, 0.2, 0.3, 0.4, and 0.5) assuming a neat hydrogel.^57^ Given the uncertainty on input properties and lack of experimental data for corresponding material’s properties, the model assumes linear elasticity. The convergence study supports its ability to provide insights within the limits of this assumption, but future work should include experimental validation.

## Results and Discussion

### Morphological and Structural Analysis

The membrane is designed to have a porosity of 5%, pore diameter of 10 μm, and thickness of 70 μm. The SEM of the top and cross section of the 3D-printed membrane is shown in Figure 11a. The top and bottom of the membrane are defined based on the printing process in the printing cell which starts from bottom and moves toward the top (see section Membrane Printing Process). The membrane shows a uniform pore distribution with 256 pores on every tile and a total of 64 tiles, leading to a membrane size of 1 × 1 cm^2^ with 16384 pores in total (see SI Figure S9a for the SEM of the top surface only). Small ripples are observed on the membrane surface in Figure 11a which can be explained based on the presence of small air bubbles. These bubbles have been either already in the cell or drawn inward because of a decrease in the volume of the printed tiles.^58^ This can further inhibit the polymerization at the end of the printing process, i.e., the membrane top surface. It is worth noting that neither the bubble nor the associated ripples affect the membrane printing process and thus the membrane porosity and/or pore size. The SEM image of the top surface of the commercial hydrophilic PTFE membrane is shown in Figure 11b, demonstrating a fibrous membrane with elliptical surface pores with a long diameter of around 10 μm, which is reported by the manufacturer^59^ (see SI Figure S9b,c for the SEM images of the bottom and cross section of the commercial PTFE membrane).

**Figure 11:**
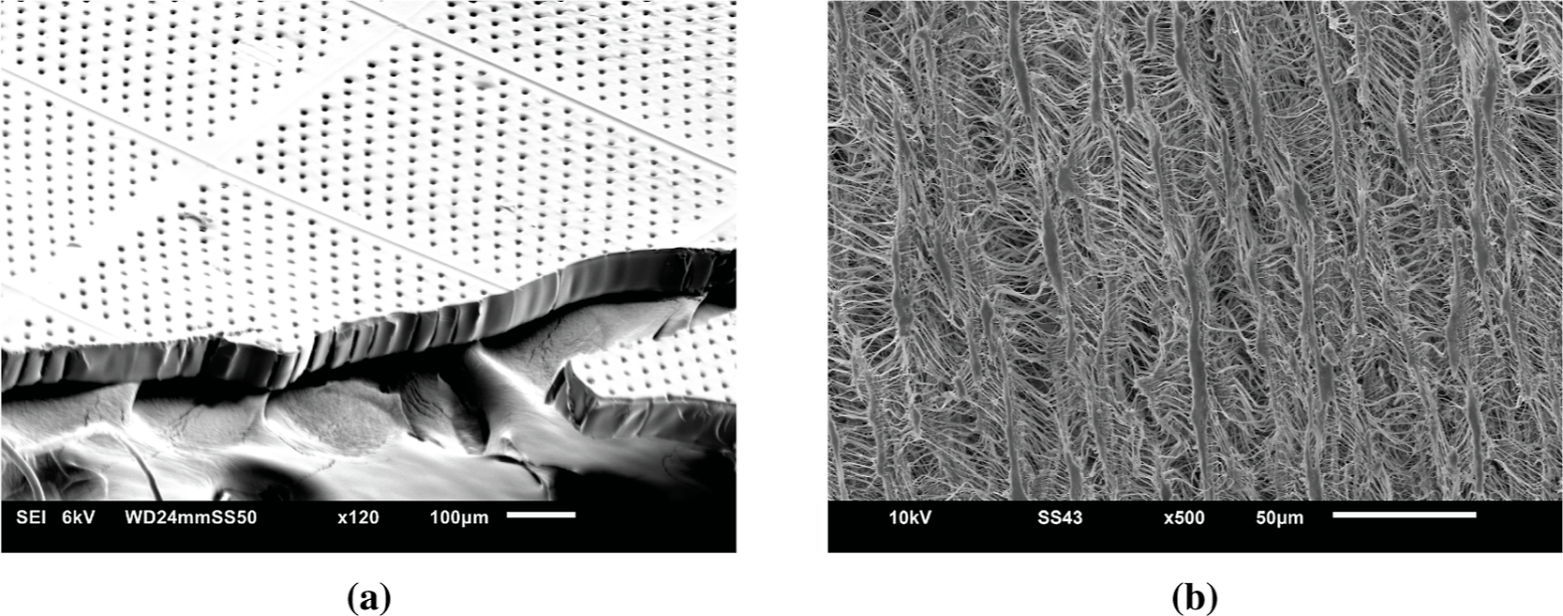
SEM images of the (a) top surface and cross section of the 3D-printed membrane with an average pore diameter of approximately 10 μm and (b) top surface of the commercial PTFE membrane, showing elliptical surface pores with a long diameter of approximately 10 μm).

The pore size distribution of the 3D-printed membrane, measured via image analysis of membranes’ SEM images (see the detailed analysis procedure in the Supporting Information section “Pore size distribution measurement: 3D-printed membrane”), is shown in Figure 12a. A uniform pore size distribution with an average pore diameter of 10.5 ± 0.32 μm is obtained. The coefficient of variation (CV) (standard deviation divided by average) is 3%, showing a narrow pore size distribution with uniform pore size (CV values smaller than 25% show monodispersity in the measured size distribution^60^).

**Figure 12:**
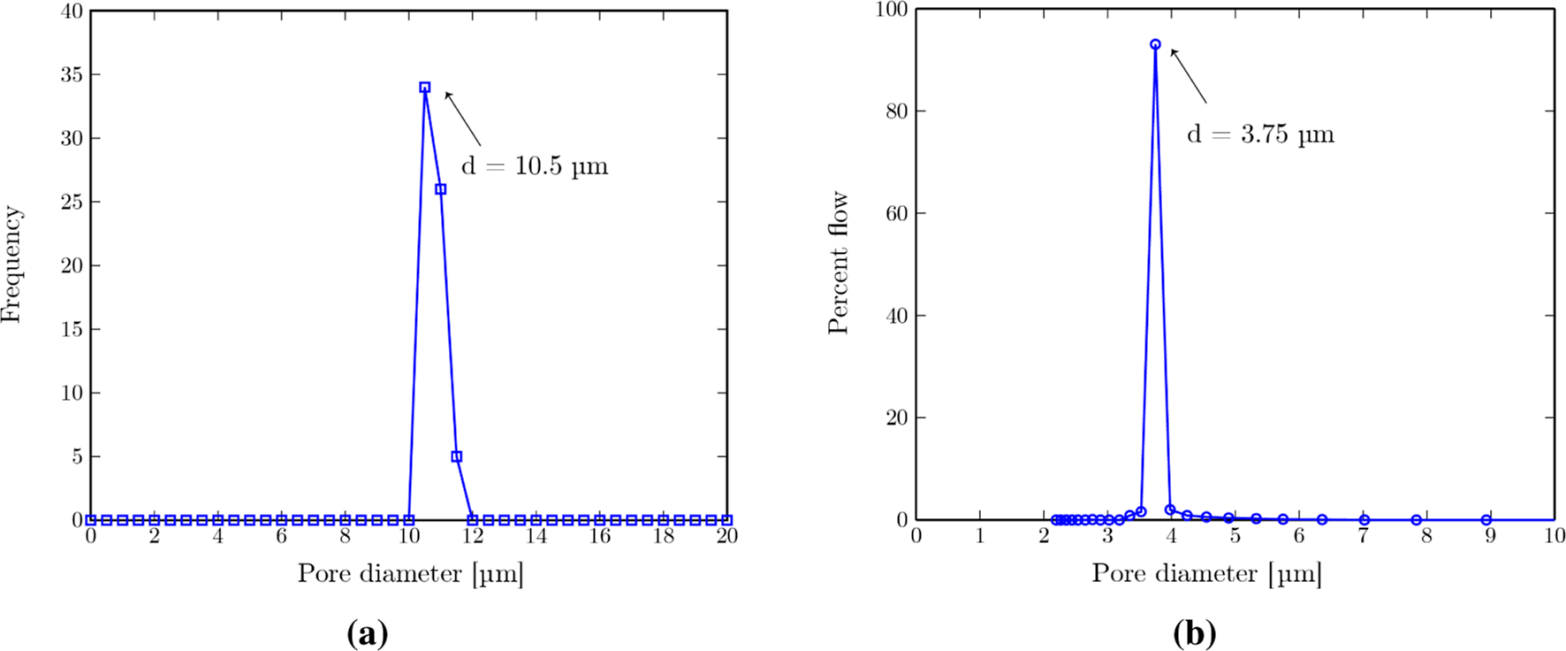
Pore size distribution of the (a) 3D-printed membrane (obtained via image analysis of the SEM images), showing a uniform pore size distribution of 10.5 ± 0.32 μm, and (b) commercial PTFE membrane (obtained via capillary flow porometry), showing an average pore diameter of 3.75 μm with a size distribution of 0.23 μm.

A 5% error margin is observed in the final pore radius of the printed membrane compared to the designed radius. This discrepancy can be caused by the low precision of the projected area, which can lead to overpolymerization at the edge of some pores. The membrane porosity (ϕ_3Dp_) is calculated as 4.7% using

4 $$\phi_{3Dp}=\frac{n\pi r^{2}}{A}$$

where *n* is the total number of pores (16384), *r* is the average pore radius of the 3D-printed membrane (5.25 μm), and *A* is the total membrane area (3.03 × 10^–5^ m^2^).

The pore size distribution of through pores in the commercial PTFE membrane is obtained via capillary flow porometry (Figure 12b), showing an average pore diameter of 3.75 μm and size distribution of 0.23 μm (full width at half max/2). Membrane porosity is further obtained as 45%, which is the percentage of the total through pore area. See the details of the measurement procedure in the Supporting Information section “Pore size distribution measurement: Commercial PTFE membrane”.

### Contact Angle and Surface Roughness

The mean static contact angle (mSCA) values of water and oil (*n*-hexadecane) in air along with those of oil and air in a water medium for commercial PTFE and 3D-printed PEGDA membranes are shown in Table 1. Both membranes show hydrophilic and underwater-oleophobic wetting properties suitable for oil removal from an oil-in-water emulsion. The lower mSCA value of water in air and higher mSCA values of air in water on 3D-printed PEGDA membranes compared to those on commercial PTFE membranes demonstrate higher hydrophilicity of the PEGDA membrane. A higher mSCA value of oil in water on the PEGDA membrane in comparison to that of the commercial PTFE membrane shows more oleophobicity of the PEGDA membrane underwater. The contact angle of oil in air cannot be measured as oil spread on both surfaces quickly. This can be explained based on the low surface tension of *n*-hexadecane (≈25 mN/m),^49^ leading to its spontaneous imbibition into the porous membranes and thus not-measurable contact angles.^61,62^

**Table 1:** Mean Static Contact Angles (mSCA) Values of 3D-Printed (3Dp) and Commercial PTFE (JCWP14225) Membranes at Different Test Conditions^a^

condition	mSCA [°]	
	3Dp membrane	commercial membrane
oil-in-water	131.96 ± 2.14	111.68 ± 2.97
air-in-water	134.15 ± 2.41	114.63 ± 4.22
water-in-air	45.54 ± 2.48	67.7 ± 1.13
oil-in-air	N.A	N.A

a The values after ± show the corresponding standard deviation.

The images of the mSCA in various conditions on both 3D-printed PEGDA and commercial PTFE membranes are shown in Figure 13.

**Figure 13:**
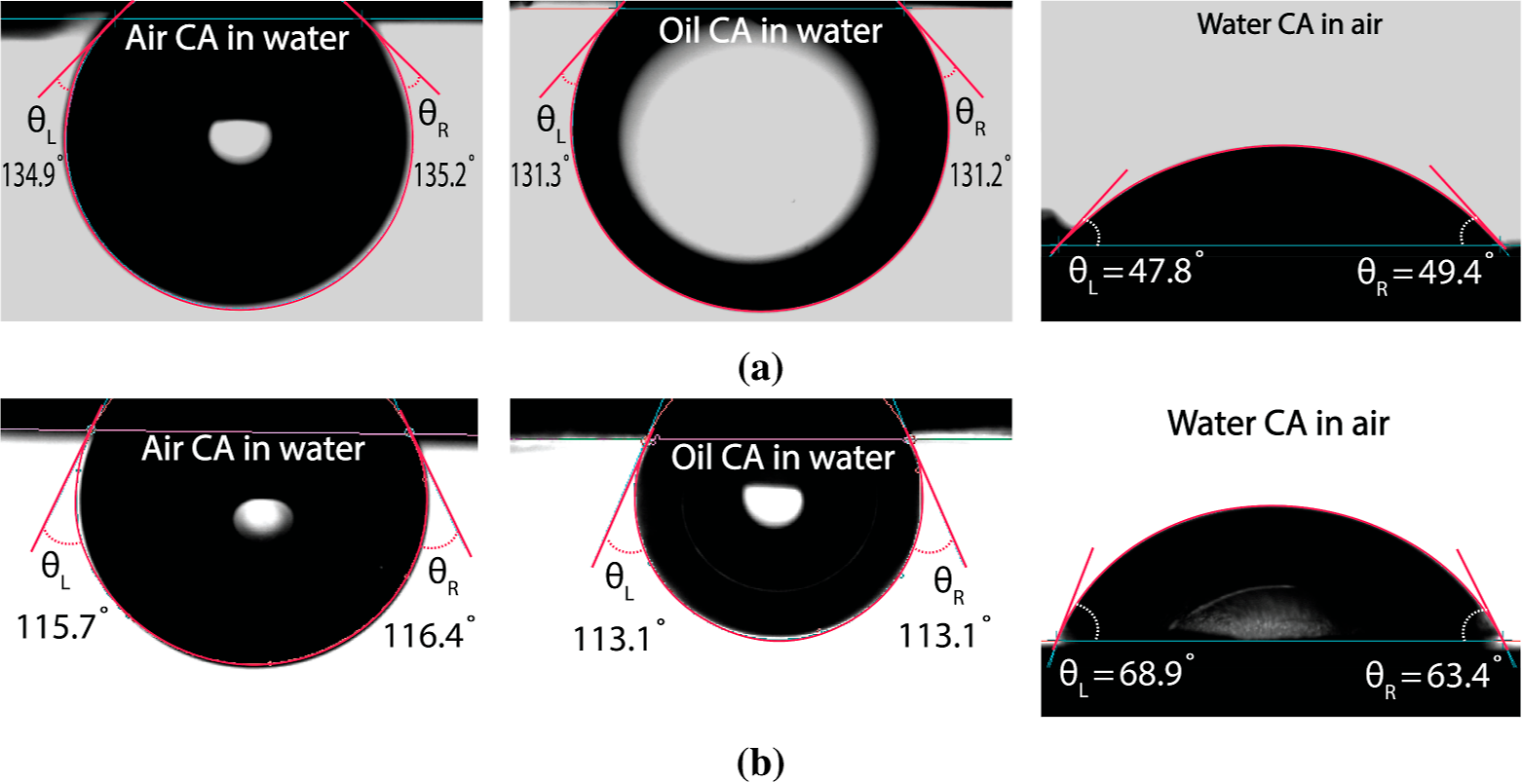
Mean static contact angles of different liquids in various environments (labeled in the figures) on the (a) 3D-printed PEGDA membrane and (b) commercial PTFE membrane. The contact angle of oil (*n*-hexadecane) in air on both membranes is not included since it was not measurable due to its low surface tension, leading to spontaneous imbibition.

The values of the advancing, receding contact angle (ARCA) and contact angle hysteresis (difference between advancing and receding CAs) of water in air on both membranes are shown in Table 2. Due to the slow water adsorption by the membranes, a drop in both advancing and receding contact angles within each cycle was observed, leading to higher standard deviation for CA hysteresis^63^ (see an example plot of ARCA in the SI, Figure S10). The 3D-printed membrane shows higher hysteresis (41.69 ± 0.90°) than that of the commercial PTFE membrane (36.66 ± 1.04°), indicating a larger surface roughness.^64^

**Table 2:** Dynamic Contact Angle Measurements of Water in Air on 3D-Printed and Commercial PTFE Membranes^a^

membrane	advancing CA [°]	receding CA [°]	hysteresis [°]
commercial	71.60 ± 1.25	34.93 ± 2.25	36.66 ± 1.04
3D-printed	59.85 ± 1.72	18.16 ± 2.42	41.69 ± 0.90

a The values after ± show the corresponding standard deviation due to averaging.

AFM was used to study membrane surface roughness and further understand the difference between contact angle hysteresis of membranes. The AFM images of both membranes are shown in SI Figure S11. The root-mean-square values, showing the surface roughness, are 451 nm for 3D-printed and 252 nm for commercial PTFE membranes. The 3D-printed PEGDA membrane shows a rougher surface than the commercial membrane, producing a more hydrophilic and underwater-oleophobic surface (see Table 1). Pronounced hydrophilicity and larger contact angle hysteresis are generally observed on surfaces with more surface roughness on a nanometer scale.^64,65^ This further demonstrates the applicability of the 3D-printed membrane for oil separation from oil-in-water emulsions.

### Water Uptake and Hydrolysis of PEGDA in Water

The wet and dry thickness of both membranes along with the corresponding water uptake ratios, calculated using eq 3, are shown in Table 3. Both membranes showed a similar degree of water uptake consistent with their hydrophilic wetting property (see section Contact Angle and Surface Roughness).

**Table 3:** Wet and Dry Thickness of Both 3D-Printed and Commercial PTFE Membranes Along with the Corresponding Water Uptake Ratios (WURs)^a^

membrane	dry thickness [μm]	wet thickness [μm]	WUR [ %]
commercial	60.4 ± 1.7	73.3 ± 2.6	21.3 ± 0.9
3D-printed	58.5 ± 1.1	70.4 ± 1.4	20.3 ± 0.2

a The values after ± are the standard deviations.

The material of the 3D-printed membrane (PEGDA) is susceptible to hydrolysis in case of long exposure to a water-based medium. In an environment with high water content, the ester bonds in PEGDA can hydrolyze, causing the monomer chains to break and degrade the membrane.^66^ This effect on the PEGDA 3D-printed membrane was investigated using FTIR measurements. The IR spectrum of the dry PEGDA and that of the polymer stored in water for 2 weeks and 3 months is shown in SI Figure S12. The detailed analysis of the FTIR peaks along with the hydrolysis reaction mechanism of PEGDA is shown in the Supporting Information, section “FTIR results”.

The FTIR analysis suggests no significant PEGDA hydrolysis during the experiments (permeability and separation), including the 1 h pretreatment step in water.

### Permeability Measurements

The pure water permeability of both 3D-printed and commercial membranes is calculated using Darcy’s law^67^

5 $$Q=\frac{\kappa A}{\mu }\frac{dp}{dx},$$

where *Q* is the total volumetric flow rate of permeating fluid (water) (m^3^/s), κ is the membrane permeability (m^2^), *A* is the total membrane area (m^2^), μ is the viscosity of permeating fluid (Pa·s), and $\frac{dp}{dx}$ is the pressure gradient across the membrane, which is considered as Δ*p* divided by the membrane thickness (*L*) (Pa/m). The wet thickness values of both membranes (see Table 3) are used to calculate the pressure drop across the membrane.

By plotting the transmembrane flux of the permeating fluid (Q/A (m^3^/m^2^·s)) multiplied by its viscosity as a function of the applied pressure gradient, the permeability κ can be directly calculated from the slope (Figure 14a). The values of membrane permeability are shown in Table 4. Membrane permeability κ can be related to porosity (ϕ) and pore radius *r* (m) according to the one-dimensional (1D) tube model (see Supporting Information, section “Relation between permeability and porosity” for derivation)

6 $$\kappa =\frac{\phi r^{2}}{8}$$

**Figure 14:**
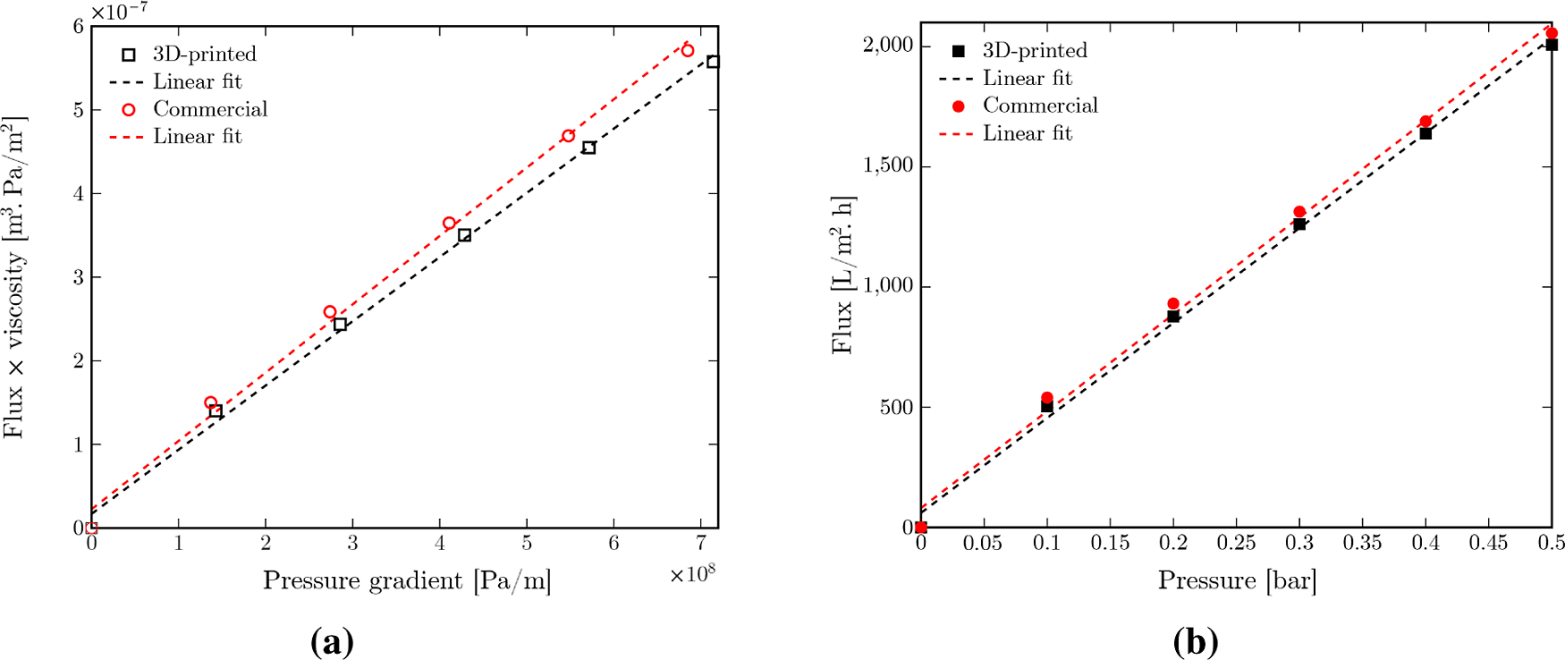
(a) Transmembrane flux × viscosity as a function of the pressure gradient for 3D-printed and commercial PTFE membranes with the slope demonstrating the permeability values (κ (m^2^)). (b) Transmembrane flux of pure water permeating through the 3D-printed and commercial membranes as a function of pressure with the slope demonstrating the permeance values (Pe (L/m^2^ h bar)). The dashed lines in both plots show the linear fit to the data points. The corresponding 95% confidence interval values are shown in Table 4.

**Table 4:** Pure Water Membrane Permeance (Pe) and Permeability (κ) for 3D-Printed (3Dp) and Commercial PTFE Membranes (JCWP14225)^a^

membrane	Pe [L/m^2^ h bar]	κ [m^2^]	κ [Darcy^b^]
3Dp	3949 ± 293.5	7.68 ± 0.57 (×10^–16^)	7.78 ± 0.58 (×10^–4^)
commercial	4030 ± 390.5	8.17 ± 0.79 (×10^–16^)	8.28 ± 0.80 (×10^–4^)

a The values after ± are half of the 95% confidence interval (CI) bounds of the linear fit ((upper CI -lower CI)/2).

b 1 Darcy = 0.987 × 10^–12^ m^2^.

Despite different pore radius and porosity, both 3D-printed and commercial membranes showed close permeability values $\left(\frac{\kappa_{3Dp}}{\kappa_{Com}}=0.94\right)$ . According to the 1D tube model (eq 6), this ratio is directly proportional to the porosity ratio $\left(\frac{\phi_{3Dp}}{\phi_{Com}}\right)$ multiplied by the ratio of the pore radius to the power two $\left({\left(\frac{r_{3Dp}}{r_{Com}}\right)}^{2}\right)$ . Using the average pore radius (see Figure 12) and the porosity ratio of both membranes (see section Morphological and Structural Analysis for measured porosity values), the permeability ratio is calculated as 0.82. The 12.8% discrepancy between calculated and measured permeability ratios can be attributed to the simplified model assumption that pores are straight cylinders (with tortuosity factor τ = 1) and oriented perpendicular to the membrane surface (similar to 3D-printed membranes). In the commercial membrane, however, pores are tortuous and oriented at random angles across the membrane, leading to smaller permeability values than those predicted by the model.

To better compare the membrane permeability results to those from typical microfiltration membranes, the thickness effects should be excluded. To do so, membrane permeance or pressure-normalized flux can be calculated. The transmembrane flux of water permeating through the membrane (J) (L/m^2^·h) is related to the driving force (applied pressure) (Δ*p*) (bar) using

7 J=Pe×Δp

where Pe is the membrane permeance or pressure-normalized flux (L/m^2^·h.bar). The plot of flux as a function of pressure for both 3D-printed and commercial membranes is shown in Figure 14b, where the slope is the permeance. The calculated values of the membrane permeance, based on the linear fit to the data, are shown in Table 4. Both membranes show comparable permeance values to those from typical microfiltration membranes (≈3800 L/m^2^.h.bar).^68^

### Separation Performance

Diluted O/W emulsions made via dispersing hexadecane oil droplets in aqueous solutions of SDS50 and SDS100 were used to study separation performance of both 3D-printed and commercial PTFE membranes (see section Separation Experiments). The oil concentration and droplet size distribution of both feeds are shown in Table 5 and Figure 15. The oil concentration in the permeate obtained from 3D-printed and commercial membranes and corresponding rejection ratios are shown in Table 6. The microscopy images of the permeates from both membranes are shown in the SI, Figures S14 and S15.

**Table 5:** O/W Emulsion Feeds, Corresponding Oil Concentration, Average (*d*_ave_), and Minimum (*d*_min_) and Maximum (*d*_max_) Droplet Diameter Values Obtained from Analyzing Five Feed Samples for Each SDS Concentration^a^

feed	oil conc. [ppm]	*d*_ave_ [μm]	*d*_min_ [μm]	*d*_max_ [μm]
SDS50	937.4 ± 275.6	14.3 ± 1.3	10.2	21.2
SDS100	569.8 ± 129.9	11.1 ± 0.9	8.1	13.6

a The values after ± are the standard deviations.

**Figure 15:**
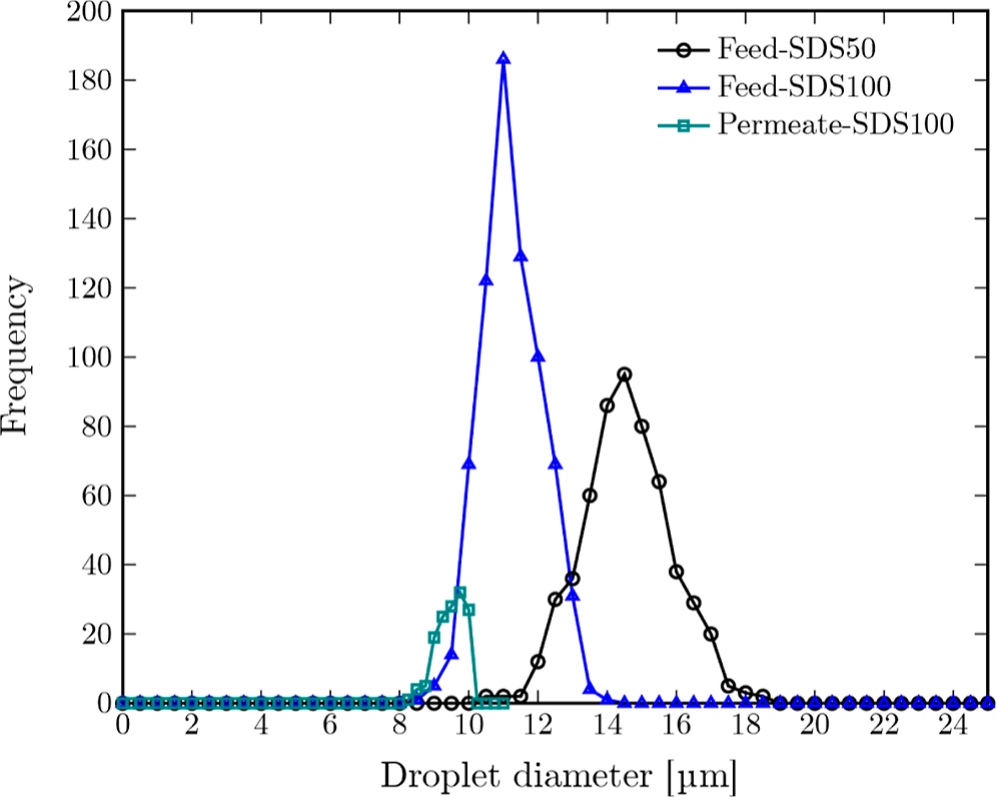
Oil droplet size distribution in feed O/W emulsions containing SDS50 and SDS100 aqueous solutions and that of the permeate from SDS100 O/W emulsion through the 3D-printed membrane. The droplet size distributions are obtained from analyzing five samples for each SDS solution.

**Table 6:** Oil Concentration in the Permeates Obtained from 3D-Printed and Commercial Membranes and Corresponding Rejection Ratios^a^

permeate	membrane	oil concentration [ppm]	rejection ratio [ %]
SDS50	3D-printed	0.0	100
	commercial	0.0	100
SDS100	3D-printed	57.2 ± 7.8	90
	commercial	0.0	100

a The value after ± shows the standard deviation obtained from analyzing five samples.

The commercial membrane showed 100% rejection of oil droplets from both feeds since its average pore diameter (3.75 ± 0.23 μm) is much smaller than the average droplet diameter in both feeds. The 3D-printed membrane could successfully retain all the oil droplets in the SDS50 feed, resulting in a rejection ratio of 100%. The rejection ratio of the 3D-printed membrane was slightly decreased to 90% while separating oil droplets from the SDS100 feed emulsion. This is mainly due to the proximity of the average droplet diameter to the average pore diameter of the membrane (10.5 ± 0.32 μm) and presence of droplets smaller than this average pore diameter. The droplet size distribution plot of both feeds and that of the permeate through the 3D-printed membrane is shown in Figure 15.

### Simulation Results

The pressure variation throughout the entire fluid domain is shown in Figure 16a, depicting a continuous transition from high pressure at one extremity to low pressure at the other. The pressure variation through the filter is obtained by slicing the domain along its depth and averaging the pressure values for each slice. The results (Figure 16b) showed that pressure drops mainly occur within the membrane region in a linear fashion. The velocity profile inside the fluid domain is observed by looking at the cross section of the membrane pores (Figure 16c), showing its parabolic nature.

**Figure 16:**
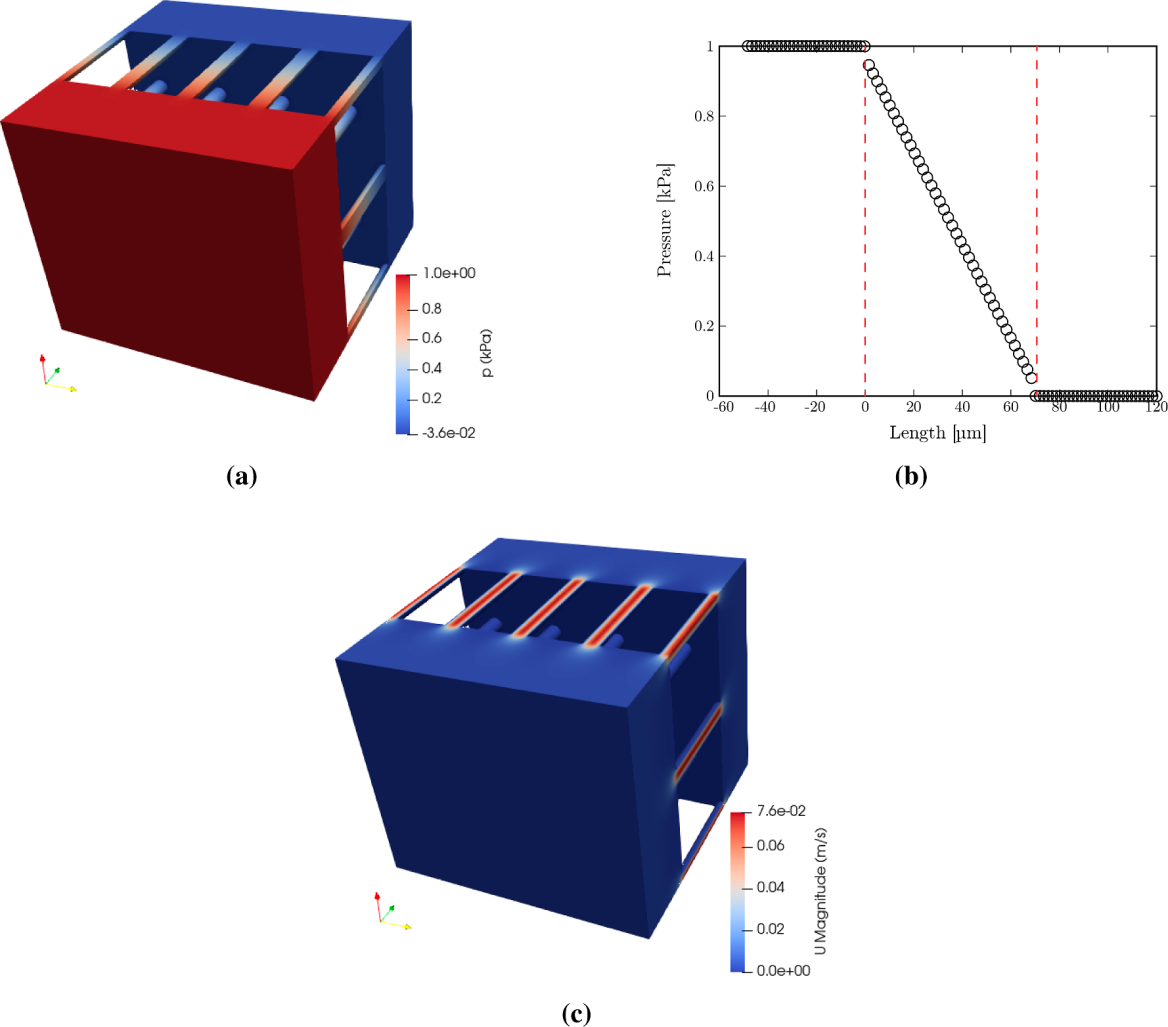
(a) Pressure field throughout the fluid domain. (b) Pressure variation along the depth of the fluid domain. The dashed lines show the membrane region with a thickness of 70 μm. (c) Velocity profile at a cross section taken across the fluid domain.

The permeability of the membrane is calculated using the integrated form of Darcy’s law (eq 5) via the following relation

8 $$\kappa =\frac{\mu Q}{\Delta p}\frac{L}{A}$$

in which Δ*p* is the pressure drop across the unit cell, *L* is the length (e.g., thickness), and *A* is the cross-sectional area. *L* and *A* are known from the unit cell geometry, fluid flow rate (*Q*) is extracted from the simulation results, and Δ*p* is evaluated using the pressure values at the limits corresponding to the membrane thickness (Figure 16b).

The 3D-printed membrane’s permeability from the simulation results is found to be κ = 1.39 × 10^–13^ m^2^, which is 3 orders of magnitude higher than the experimentally measured values (Table 4). The potential origin of this discrepancy has been further explored considering pore deformation/compression.

Since the PEGDA material of the 3D-printed membrane is a hydrogel, it can undergo significant deformation during pressurization (see section Pore Deformation for the simulations details). In the case where the membrane is free to expand (case b), the pore diameter increases with swelling and the applied pressure due to the unconstrained condition. However, the membrane is constrained during the experiments (see Figure 7) and the constrained case (case a) should be considered. In this case, the membrane pore shows a significant reduction in diameter. The cumulative distribution of the relative changes in pore radius at the inlet and outlet of the geometry (Figure 10) is shown in the SI, Figure S16. A wide range of deformations (pore reduction and expansion) may occur in the pore depending on the conditions. Due to the pressure gradient along the pore length, the deformation is different at the inlet, where the pore tends to expand, and at the outlet, where it reduces, resulting in a diameter gradient along the pore length. Figure 17 shows a typical profile of a deformed pore. In this case, the pore radius is reduced at one end and expanded at the other. Notably, the pore deformation leads to pore buckling in many cases. The simulations are stopped at the buckling point, and the postbuckling behavior is not considered.

**Figure 17:**
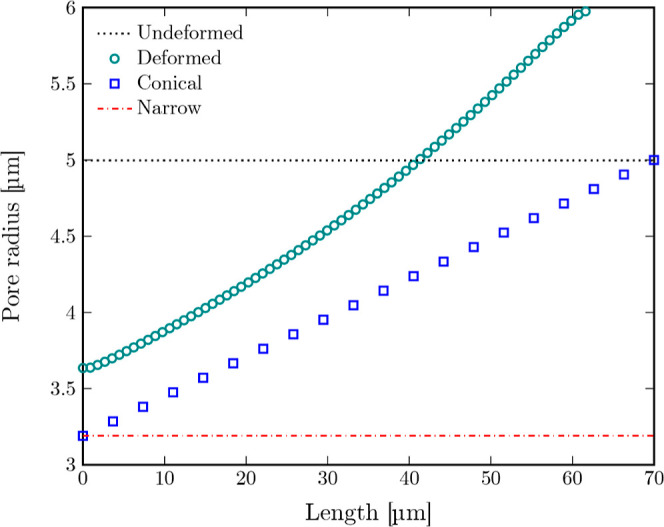
Different pore profiles along the depth of the simulation unit cell. The deformed curve exhibits the typical profile of a pore after pressurization. The narrow profile (dash-dotted red line) corresponds to the minimum radius of 3.19 μm obtained from the simulations and corresponds to a worst-case configuration for permeability. Based on that, a conical pore profile is used to approximate a worst-case deformed pore through linear interpolation between the minimal and undeformed pore radius.

A deformed pore shape gives rise to different velocity profiles, resulting in different flow rates (*Q*), as shown in Figure 18. The computed permeability values for the deformed, narrow, and conical pore shapes are κ = 9.19 × 10^–14^ m^2^, κ = 2.33 × 10^–14^ m^2^, and κ = 5.20 × 10^–14^ m^2^, respectively. As expected, the narrow pore shape results in the lowest permeability, an order of magnitude lower than the value computed with the undeformed shape (κ = 1.39 × 10^–13^ m^2^). Nevertheless, this permeability value remains approximately 2 orders of magnitude higher than the experimental ones.

**Figure 18:**
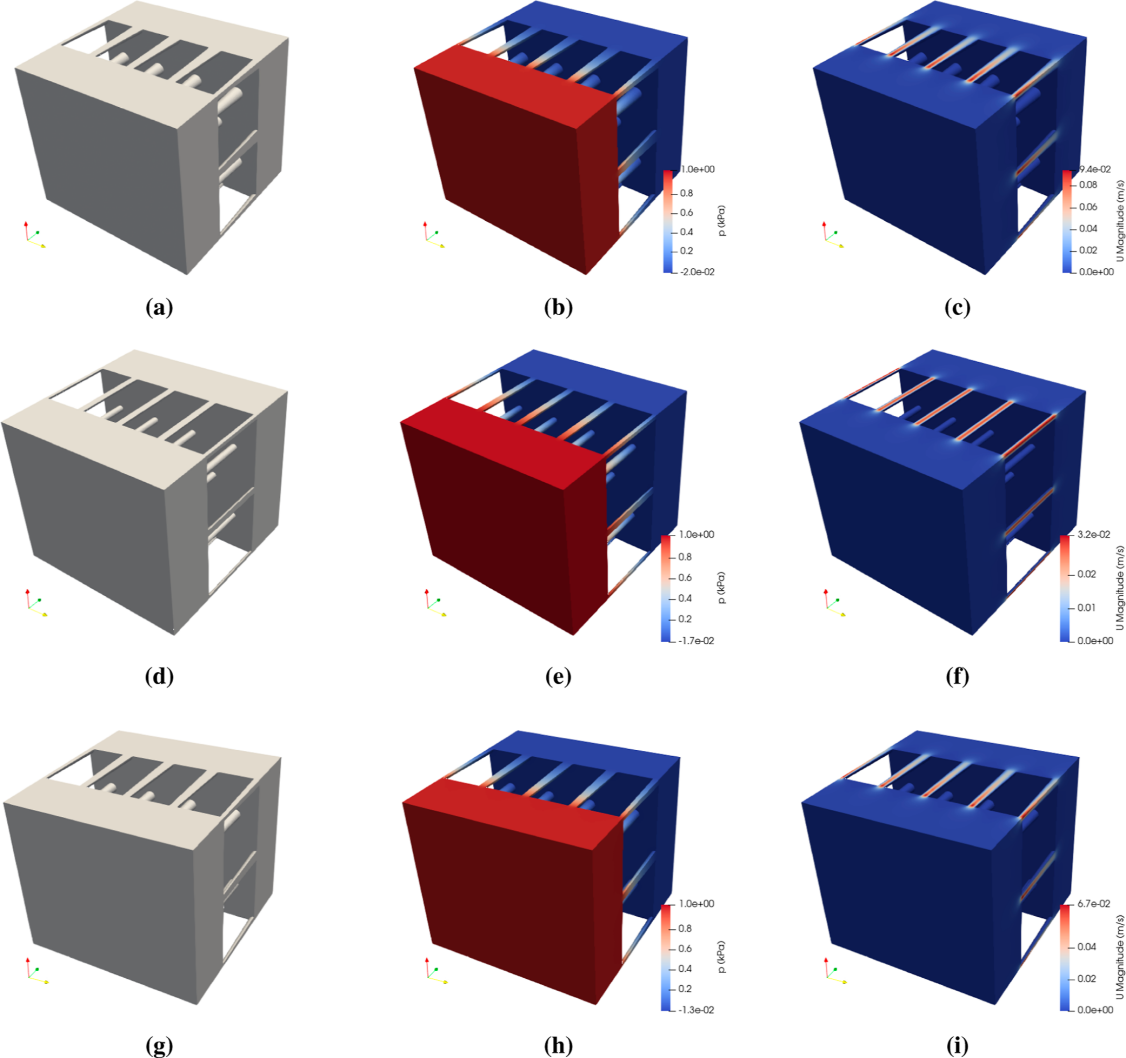
(a) Reduced unit cell with a deformed pore profile (teal circles in Figure 17); the corresponding pressure and velocity fields are, respectively, shown in (b) and (c). (d) Reduced unit cell with a narrow pore profile (red dash-dotted line in Figure 17); the corresponding pressure and velocity fields are, respectively, shown in (e) and (f). (g) Reduced unit cell with a conical pore profile (blue squares in Figure 17); the corresponding pressure and velocity fields are, respectively, shown in (h) and (i).

To better understand the discrepancy between the experimental and simulated permeability values, the theoretical pore radius, corresponding to the experimentally measured permeability, is back-calculated using the 1D tube model (eq 6), considering the geometry in Figure 10. The porosity ϕ is the ratio between the pore area π*r* ^2^ and total area *A* (ϕ = π*r* ^2^/*A*). Considering the initial pore diameter of 10 μm and porosity of 5%, the total area can be calculated as *A* = 1.57 × 10^–9^ m^2^. Substituting the relation for porosity (ϕ = π*r* ^2^/*A*) into eq 6 gives κ = π*r* ^4^/8*A*. Using the calculated value of *A* = 1.57 × 10^–9^ m^2^, the pore radius is computed to be 1.32 μm. The back-calculated pore size results in a corresponding porosity of 0.35% and a pore radius reduction of 73.6%, significantly larger than the ones obtained by numerical modeling. This, however, indicates that a pore deformation may indeed cause such results.

Two reasons can help explain the discrepancy between the numerical and experimental permeability values. First, the properties used in the simulations are approximated from the literature. Second, the reported numerical simulations do not account for the complex multiphysics occurring in reality. For instance, a high-fidelity model should account for the equilibrium between pore deformation and fluid pressure gradient, large-scale deformation of the membrane, such as bending, and explore buckling. These limitations highlight the need for further research in modeling permeability of porous membranes.

## Conclusions

In this work, we demonstrate for the first time (to the best of our knowledge) a fully 3D-printed microfiltration membrane with pore size around 10 μm, using the dual-wavelength microstereolithography printing method.

The membrane printing procedure including the membrane design and postprocessing is thoroughly studied. A novel gradient descent method is used to calculate the projection area for printing the membrane. Using this method, we are able to successfully control the precise projection of blue light and UV light, leading to a highly controlled projection zone and thus a successful directly 3D-printed membrane. A printing cell is successfully designed and fabricated to print membranes with consistent thickness values. The heating step after membrane printing increases the membrane’s mechanical integrity, leading to reproducible membranes. The printed membranes show a narrow pore diameter of 10.5 ± 0.32 μm, close to the designed pore diameter of 10 μm.

The 3D-printed membranes are extensively characterized via SEM, FTIR, contact angle, surface roughness (AFM), and pure water permeability measurements. The separation performance is further investigated and compared with that of the commercial PTFE membrane by filtering monodisperse oil-in-water emulsions through the membranes.

Both membranes show hydrophilic and under-water oleophobic wetting properties. The rougher surface of the 3D-printed membranes on the nanometer scale, obtained via AFM, leads to a more hydrophilic behavior and larger contact angle hysteresis compared to those of the commercial PTFE membrane, demonstrating its applicability for oil separation from oil-in-water (O/W) emulsions.

The separation results demonstrate that both membranes can successfully retain oil droplets from two O/W emulsions with different surfactant concentrations and average oil droplet diameters (≈14 and 11 μm). The 3D-printed membranes show a rejection ratio of 100% for separation of oil droplets with an average diameter of 14 μm. A rejection ratio of 90% is observed while separating droplets with an average diameter of 11 μm due to the presence of smaller droplets (≈8 μm) which can pass through membrane pores of 10.5 μm in diameter.

The material of the 3D-printed membrane (PEGDA) is susceptible to hydrolysis in a water medium. The FTIR results confirm that no hydrolysis occurred during the permeability and separation experiments, including the 1 h pretreatment step in water.

Despite different pore radius and porosity values, both commercial and 3D-printed membranes show similar pure water permeability values (κ ≈8 × 10^–16^ m^2^). A simplified 1D tube model is used to predict the permeability based on porosity and pore radius. In this model, pores are assumed to be cylindrical tubes oriented at right angles, which is similar to the pore configuration in the 3D-printed membrane.

The calculated ratio of permeability values (κ_3Dp_/κ_Com_) is around 13% lower than the experimental ratio since the commercial membrane contains tortuous pores, which are oriented at random angles across the membrane. This leads to a smaller permeability value for the commercial membrane compared to the predicted one using the 1D tube model.

To better understand the discrepancy between theoretical and experimental permeability values and provide the possibility to predict the pure water permeability of the 3D-printed membrane, numerical simulations are performed using the open-source software package, OpenFOAM.

The initial results show a higher permeability than the experimental value. The influence of the material’s properties and pore deformation upon pressurization are investigated as possible reasons. The results show that swelling of the 3D-printed membrane material (PEGDA) along with the pore deformation decreases the pore size, lowering the permeability. The simulations revealed the effect of material’s property on predicting membrane’s permeability, providing a valuable tool in 3D-printed membrane design optimization.

The 3D-printed membrane with well-defined and narrow pore size distribution has great prospects in a wide range of applications, spanning (waste)­water treatment to biomedical applications. Our results showcase that the novel dual-wavelength volumetric microlithography method is a suitable technique for 3D printing membranes with similar performance as the commercial membranes. The simulations further reveal that the material property has a significant effect on predicting the membrane’s permeability, providing a valuable tool to analyze other polymer alternatives, such as polyethylene glycol diacrylamide (PEGDAA). PEGDAA may exhibit a more robust performance under long-term exposure in aqueous environments. The ester bond in PEGDAA is replaced with an amide bond, preventing hydrolysis.^69^

We believe as future improvements are made, e.g., printing membranes with cone-shaped pores from various materials, spanning different polymers, such as PEGDAA, to ceramics, this manufacturing method will become one of the go-to methods for manufacturing membranes with high selectivity and permeability.

## Supplementary Material



## Notes

Membrane design calculation and printing process; pore size distribution measurements: 3D-printed and commercial PTFE membranes; O/W emulsion preparation: concentrated feed and diluted feed; SEM images; dynamic contact angle measurement; AFM results; relation between permeability and porosity: derivation of the 1D tube model; FTIR results; and separation experiments: permeate images (PDF)
