# Supplementary material for: 3D-Printed Microfiltration Membranes via Dual-Wavelength Microstereolithography
Source: ACS Omega. 2025 Aug 21;10(34):39174–91. doi: 10.1021/acsomega.5c05746 (PMC12409533; doi:10.1021/acsomega.5c05746)
Supplement: Supplementary file 1 [file ao5c05746_si_001.pdf]

3D-printed microfiltration membranes via dual wavelength micro-stereolithography

Hanieh Bazyar,*,† Shang-Che Wu,‡ Irem Gurbuz,‡ Athanasios Papageorgiou,† Wesley van Vliet,¶ Alexander Kostenko,¶ Jimmy G. Jean,§ Guillaume Broggi,§ and Baris Caglar§

†*Transport phenomena, Chemical engineering Department, Faculty of applied sciences, Delft University of Technology, Van der Maasweg 9, 2629HZ Delft, The Netherlands.*

‡*Engineering Thermodynamics, Process & Energy Department, Faculty of Mechanical, Maritime and Materials Engineering, Delft University of Technology, Leeghwaterstraat 39, 2628CB Delft, The Netherlands.*

¶*Photosynthetic B.V., De Boelelaan 1085, 1081HV Amsterdam, Amsterdam, The Netherlands*

§*Aerospace Structures and Materials Department, Faculty of Aerospace Engineering, Delft University of Technology, Kluyverweg 1, 2629 HS Delft, The Netherlands.*

E-mail: h.bazyar@tudelft.nl

Phone: +31(0)152782760

Supporting information

Membrane design calculation and printing process

The light projection was calculated via an iterative procedure using the gradient descent method. UV and blue light projection at every pixel is calculated by considering the point spread function (psf), which is a function of numerical aperture (NA) and wavelength (λ).[1] The UV and blue light

intensities projected on the resin are corrected by multiplying the psf:

$$I_{blue,pixel} = PSF(\lambda_{blue}, NA) * I_{blue,lightsource} \tag{S1}$$

$$I_{UV,pixel} = PSF(\lambda_{UV}, NA) * I_{UV,lightsource} \tag{S2}$$

The rate of polymerization is then derived from the following equation[2]

$$R_{poly} = k(I_{blue,pixel} - \beta I_{UV,pixel})^{0.5}, \tag{S3}$$

in which R_{poly} is the polymerization reaction rate, k is the reaction rate constant, β is the inhibition coefficient, I_{blue} and I_{UV} are the blue light and UV light intensities, respectively. The reaction rate constants k and β are measured beforehand. The conversion is calculated using the polymerization reaction rate via the following equation[3]

$$C_{xyz} = \int R_{poly} dt, \tag{S4}$$

in which C_{xyz} is the conversion of a certain pixel. The conversion is then compared with the target, providing a mean square error term. Via the gradient descent method, a new set of light projections can be determined:[4]

$$I_{blue,new} = I_{blue,old} - \eta \frac{\partial C_{xyz}}{\partial I_{blue,old}} \tag{S5}$$

$$I_{UV,new} = I_{UV,old} - \eta \frac{\partial C_{xyz}}{\partial I_{UV,old}} \tag{S6}$$

in which η is a predetermined step size. The algorithm reaches a solution once the mean square error is minimized. This produced the printing model, with the information on the actual light

dosages required and the chemical conversion at each location. The light intensities are shown in Figure S1, and the chemical conversion in Figure S2. A flowchart of the procedure is also provided in Figure S3. A more detailed version of the calculation method is provided by Mulder et al.[5] and Kotsenko et al.[6]

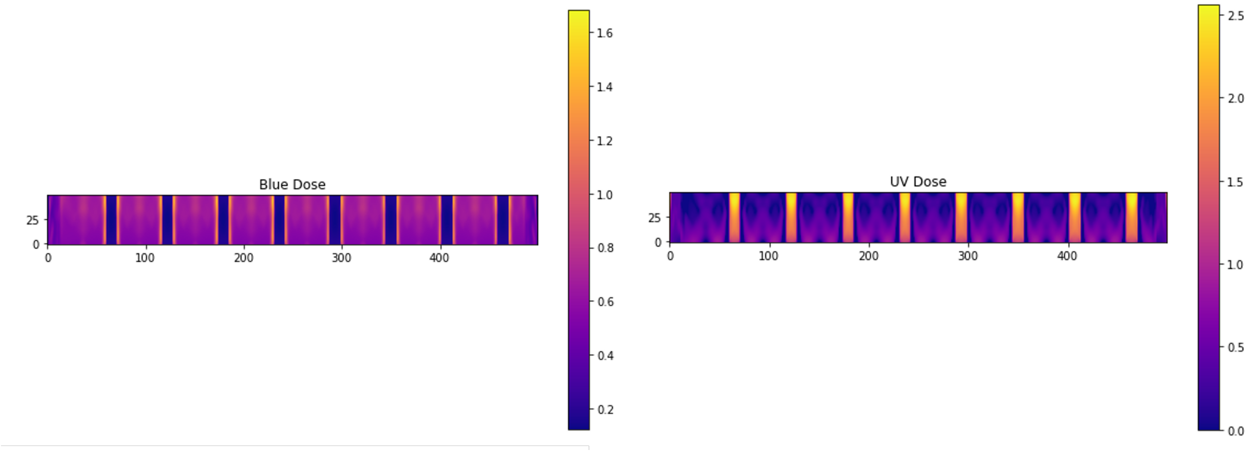

Figure S1: The dosage required for printing the membrane. The right-hand bar depicts the time amount in which the respective light source is used. The unit is in seconds (s). The x and z values are the number of pixels.

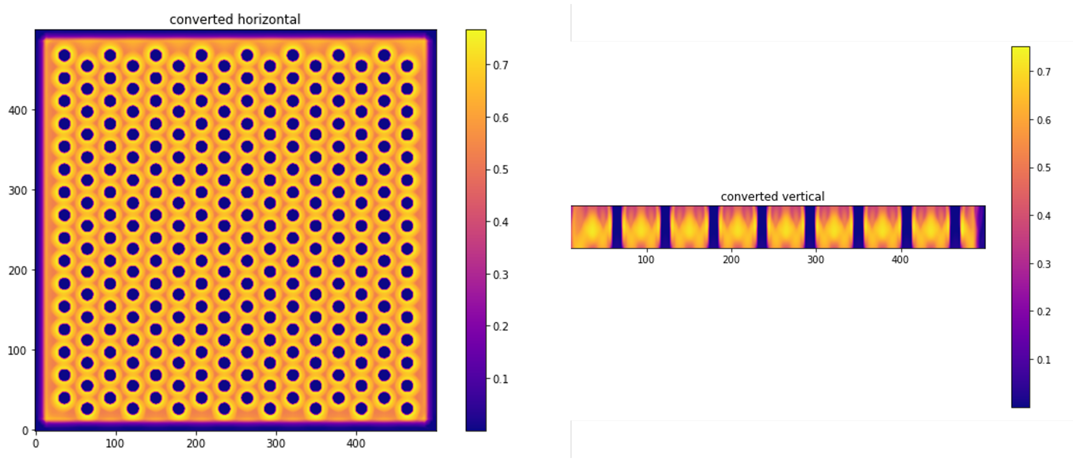

Figure S2: The final calculated conversion of the membrane, indicating the conversion rate of every pixel of the membrane, and the right-hand bar indicates conversion(%). The x, y, and z values are the number of pixels.

The schematic illustration of the printing cell with particles on the four corners of the printing zone is shown in Figure S4.

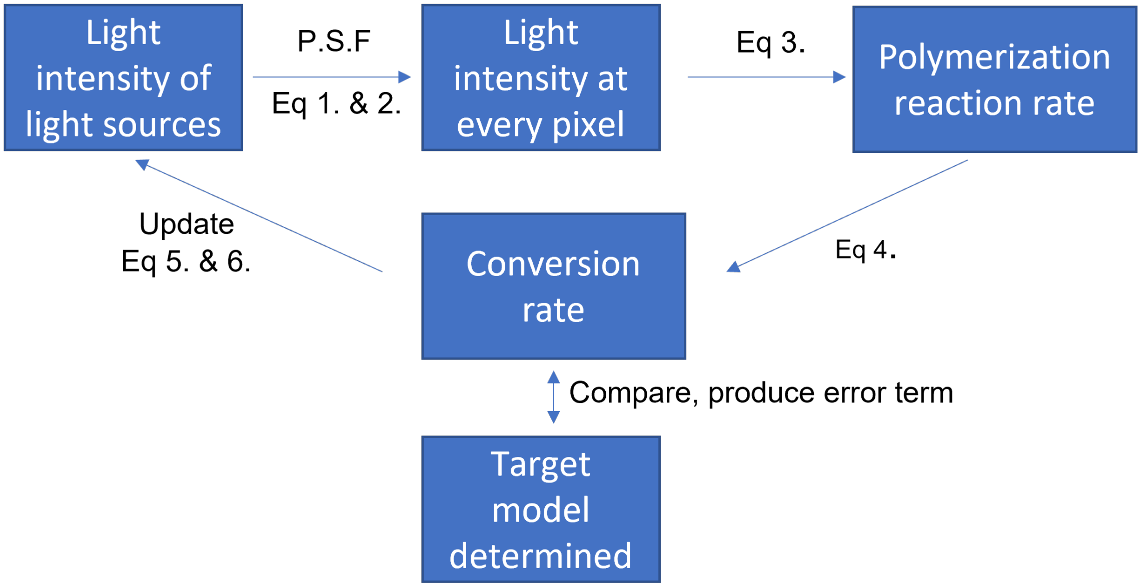

Figure S3: A flowchart showing the procedure of how the designed membrane is calculated.

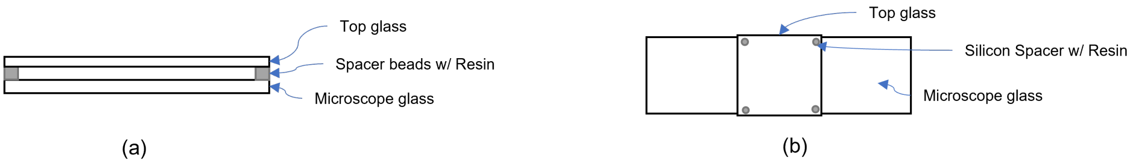

Figure S4: Schematic illustration of (a) the top and (b) the side view of the membrane printing cell.

Pore size distribution measurements

3D-printed membrane

For the pore size distribution FIJI ImageJ program[7] is utilized via importing SEM image of the membrane to the program. The image was cut to remove the SEM information bar at the lower part. The image was converted to TIFF format and the scale was set in the program. The image was binarized with a thresholding procedure and different threshold values were tested until only the pores appeared colored. Figure S5 represents the image after the adjusted thresholding procedure. 255 data sets on the 2 membranes are recorded. The pore area and subsequently diameter are measured using the function "analyze particle", leading to the corresponding pore size distribution.

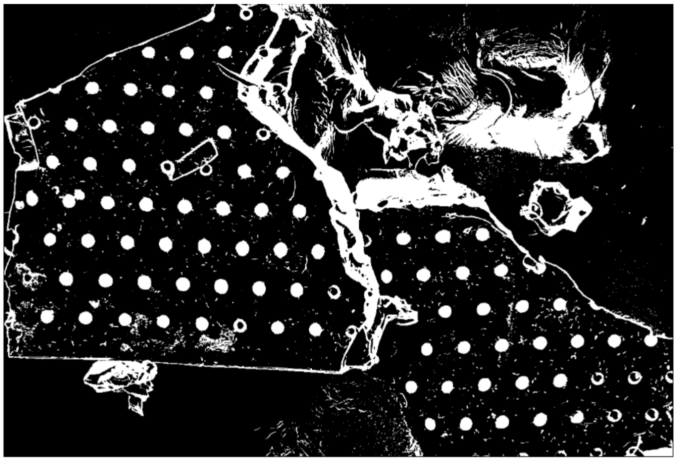

Figure S5: The SEM image of the 3D-printed membrane after thresholding in FIJI Image J.

Commercial PTFE membrane

Capillary flow porometry measurement on PTFE membrane is done by pushing nitrogen gas through the membrane infused with a low surface tension liquid, i.e. Porefil, at different pressure values. The corresponding gas flow rate is measured simultaneously. Figure S6 shows the nitrogen gas flow rate as a function of pressure for the PTFE membrane. This measurement is conducted in two runs: in the first run, the so called wet curve, a sudden increase in gas flow rate is observed which corresponds to the pressure where the biggest pores are opened (first bubble point (FBP)). In the second run as the liquid has been already pushed out, a linear behaviour between flow and pressure is obtained (dry curve). The point where wet and dry curves meet corresponds to the smallest pore size. The half-dry curve is also plotted where the flow values are half of the flow values of the dry curve. The crossing point of this curve and wet curve determines the mean flow pore size (MFP). The Young-Laplace equation

$$\Delta P = \frac{2\gamma|\cos\theta_E|}{r}, \tag{S7}$$

by considering total wetting (θ_E=0°), is used to relate pressure to the pore size. In total wetting, the liquid spreads entirely on the substrate due to the strong attraction between the liquid molecules and the solid substrate. In this case the solid-air interface is substituted completely by the solid-

liquid interface.[8] If the substrate is a porous medium, the liquid imbibes through the pores. In this equation r is the pore radius (m), γ is the interfacial tension (N/m) between permeating fluid and liquid, and θ_E is the advancing contact angle of the permeating fluid with respect to the pore wall.

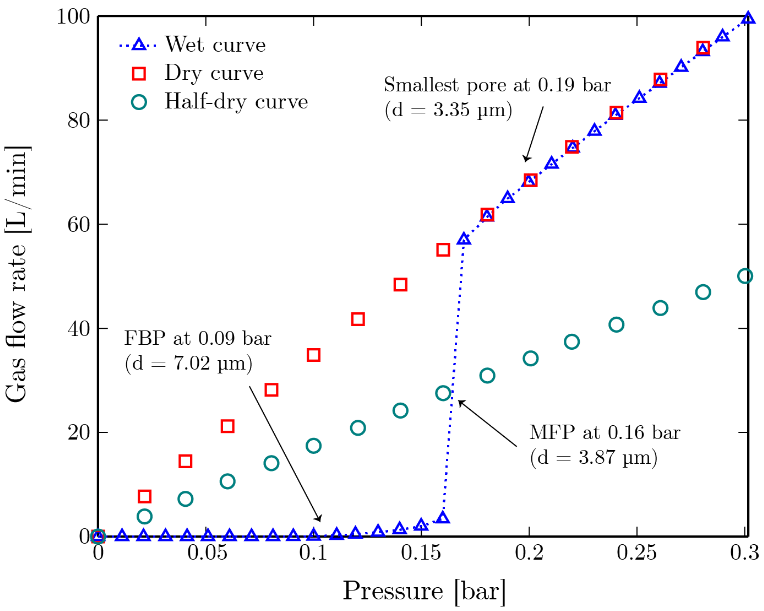

Figure S6: Capillary flow porometry results of the commercial PTFE membrane, showing flow rate of nitrogen gas as a function of pressure. The corresponding pressure values for the biggest pore size (first bubble point (FBP)), mean flow pore size (MFP), and smallest pore size are indicated with arrows.

O/W emulsion preparation

Concentrated feed

The microscopy images of the concentrated O/W emulsion in SDS50 and SDS100 aqueous solutions, obtained directly from the droplet generator are shown in Figure S7(a) and Figure S7(b). The image of a 0.2 μL of the concentrated emulsion sample is taken and fitted into a circle to measure the total radius of all the accumulated oil droplets in the sample (see Figure S7(c)). More zoomed-in images are further taken to measure the diameter of individual oil droplets, at least 10 times, to get an average droplet diameter and volume. By dividing the total area of the accumulated oil droplets by that of one droplet, the total number of droplets in the sample is calculated. The concentration of the sample in vol.% and ppm is subsequently calculated by dividing total volume

of droplets (number of droplets × average droplet volume) to that of the sample (0.2 μL). This calculation is done at least 3 times to get an average concentration for each emulsion.

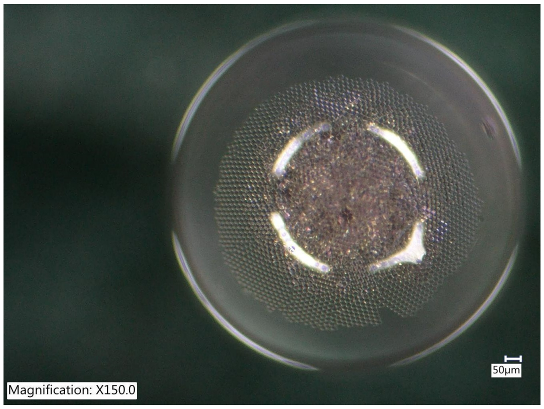

(a)

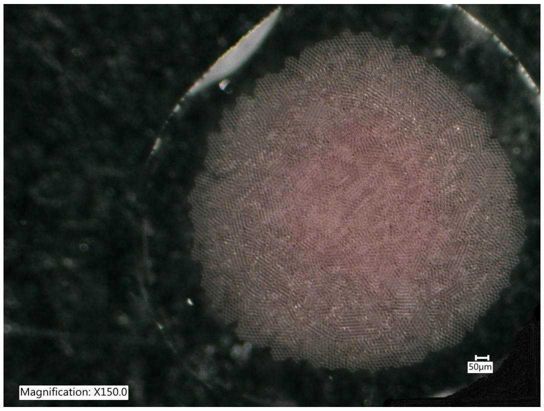

(b)

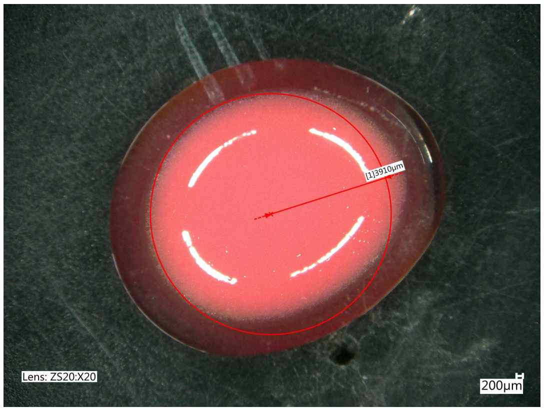

(c)

Figure S7: Digital microscopy images of the concentrated O/W emulsion fabricated by dispersing n-hexadecane oil droplets in (a) SDS50 and (b) SDS100 aqueous surfactant solution. (c) Microscopy image showing the fitted circle to the total area of the accumulated oil droplets.

Diluted feed

By knowing the initial concentration of the feed (C_i) and its volume (V_i), the required final volume for dilution (V_f) was calculated using $C_iV_i = C_fV_f$, by setting the final concentration in the diluted emulsion (C_f) to 500 ppm. The microscopy images of the diluted O/W emulsion feeds are shown in Figure S8. For consistency purposes, 0.2 μL of the feed is placed on a microscopy glass slide to take the images. For each diluted feed, at least 5 samples are taken and droplet diameter, volume, and number of droplets in each sample are measured manually in FIJI ImageJ using the

corresponding images. The data from all 5 samples is further used to calculate the total number of droplets and average droplet volume in a 1 μL sample. Due to discrepancies between the droplet size and the number of droplets in each sample, the concentration of the diluted feeds were again calculated using the procedure explained above.

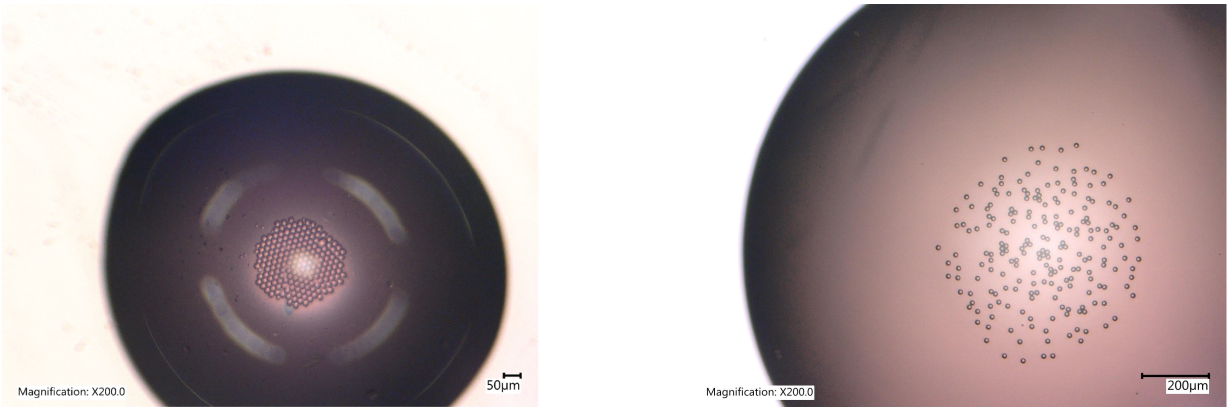

(a) (b)

Figure S8: Digital microscopy images of the diluted O/W emulsion fabricated by dispersing n-hexadecane oil droplets in (a) SDS50 and (b) SDS100 aqueous surfactant solution.

SEM images

The scanning electron microscopy images of the 3D-printed and commercial PTFE membranes are shown in Figure S9.

Dynamic contact angle measurement

The dynamic contact angle measurements were performed using advancing and receding contact angle method (ARCA) of the OCA goniometer. Advancing and receding contact angles were determined using sessile drop needle-in mode. In this mode the needle was placed in the drop and testing liquid (water) was continuously supplied or withdrawn up to 6 μL at 0.5 μL/min within six cycles. The delay time between each advancing and receding measurement was 2 s. The ARCA results of water-in-air on both membranes within the first 3 cycles are shown in Figure S10.

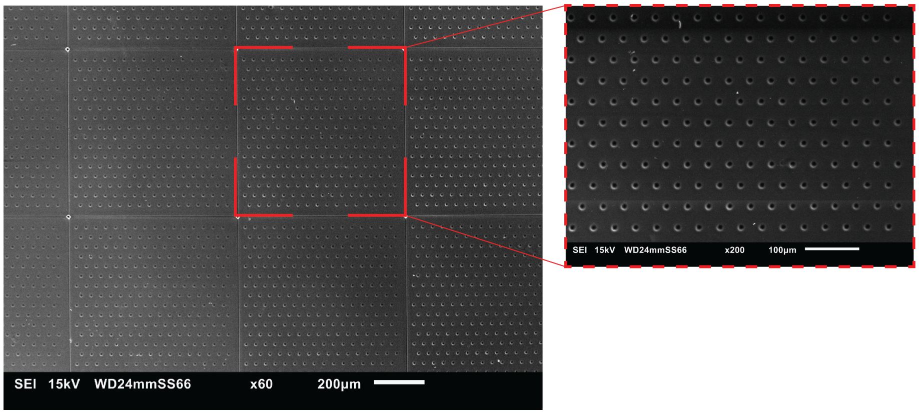

(a)

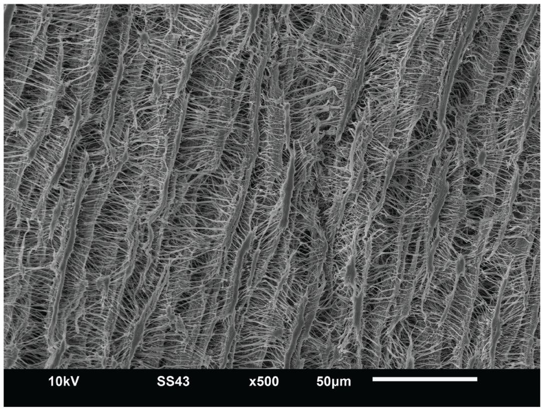

(b)

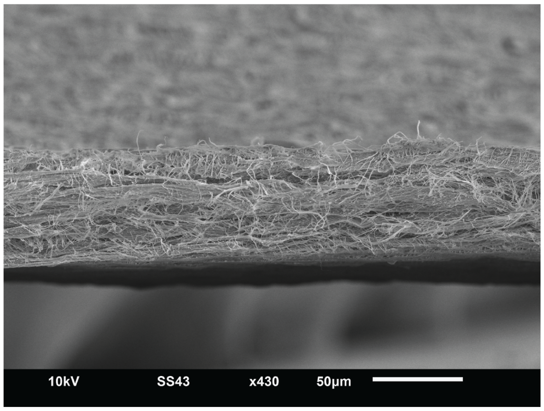

(c)

Figure S9: Scanning electron microscopy images of (a) top surface of the 3D-printed membrane, (b) bottom surface, and (c) cross section of the commercial PTFE membrane.

AFM results

The atomic force microscopy (AFM) images of 3D-printed and commercial PTFE membranes are shown in Figure S11. Both are imaged in the tapping mode on an area of $3\mu m \times 3\mu m$.

Relation between permeability and porosity

Derivation of 1D tube model

Hagen-Poiseuille equation (Equation S8) can be used to obtain the flow through a straight tube with circular cross section.[9,10]

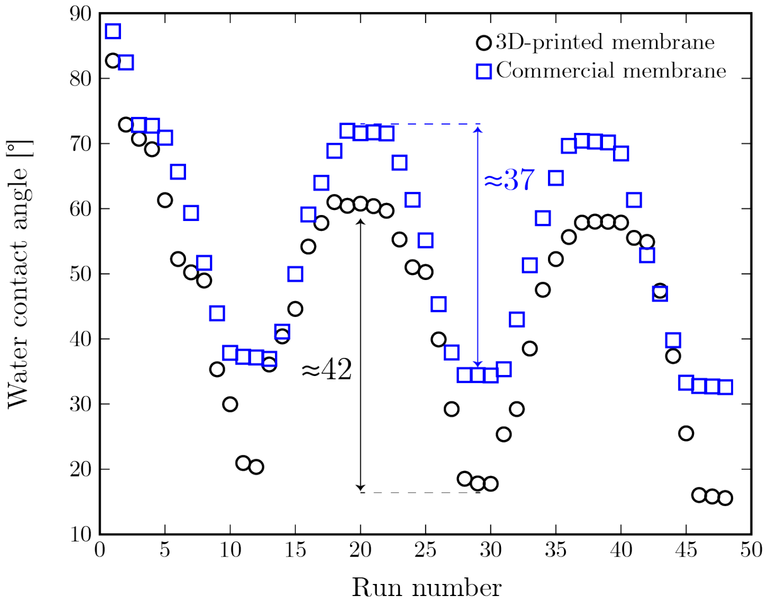

Figure S10: Advancing and receding contact angle results of water-in-air on both 3D-printed and commercial PTFE membranes within the first 3 cycles. The values next to the arrows show the average contact angle hysteresis.

$$Q = \frac{\pi r^4}{8\mu} \frac{\Delta P}{L} \tag{S8}$$

In this equation Q is the volumetric flow rate (m^3/s), r is the radius of the tube (m), μ is the viscosity of the permeating fluid (Pa s), ΔP is the pressure drop (Pa) over the length of tube L (m).

In a simple one-dimensional permeability model for a porous medium, we assume a collection of such tubes in a cubical piece with the length L. If n tubes of length L and radius r pass through the sample at right angles, the porosity ϕ reads

$$\phi = \frac{n\pi r^2}{L^2}. \tag{S9}$$

By knowing the Q through one pore (Equation S8), the total volumetric flow through the porous medium can be calculated as $Q_{total} = nQ$. By substituting $n\pi r^2$ using Equation S9, Q_{total} reads

$$Q_{total} = \frac{\phi L^2 r^2}{8\mu} \frac{\Delta P}{L}. \tag{S10}$$

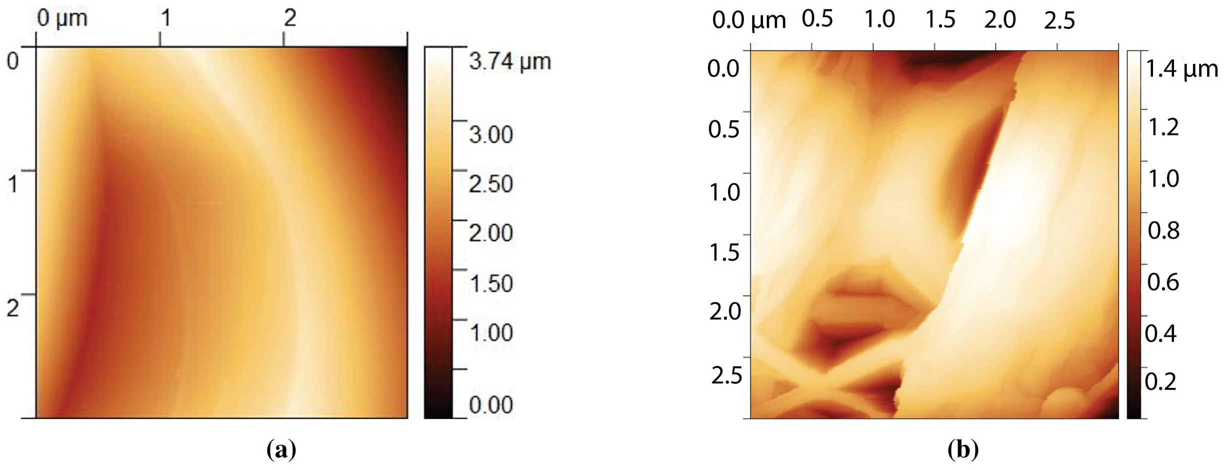

Figure S11: Atomic force microscopy (AFM) images comparing (a) 3D-printed membrane and (b) commercial PTFE membrane. The surface roughness (root mean square values) are 451 nm and 252 nm for the 3D-printed and commercial PTFE membranes, respectively.

By comparing Equation S10 with the Darcy's law[11]

$$Q = \frac{\kappa A}{\mu} \frac{\Delta P}{L}, \tag{S11}$$

where $A = L^2$ is the area of the porous medium, the relation between the permeability and porosity using this simple one-dimensional model is obtained as

$$\kappa = \frac{\phi r^2}{8}. \tag{S12}$$

FTIR results

The IR spectrum of the dry PEGDA and that of the polymers stored in water for 2 weeks and 3 months are shown in Figure S12(a). It is worth noting that for each condition three samples are tested, all showing similar IR spectra. Thus, only one spectrum is shown here. The zoomed-in IR spectrum of the PEGDA polymer sample stored in water for 3 months is illustrated in Figure S12(b).

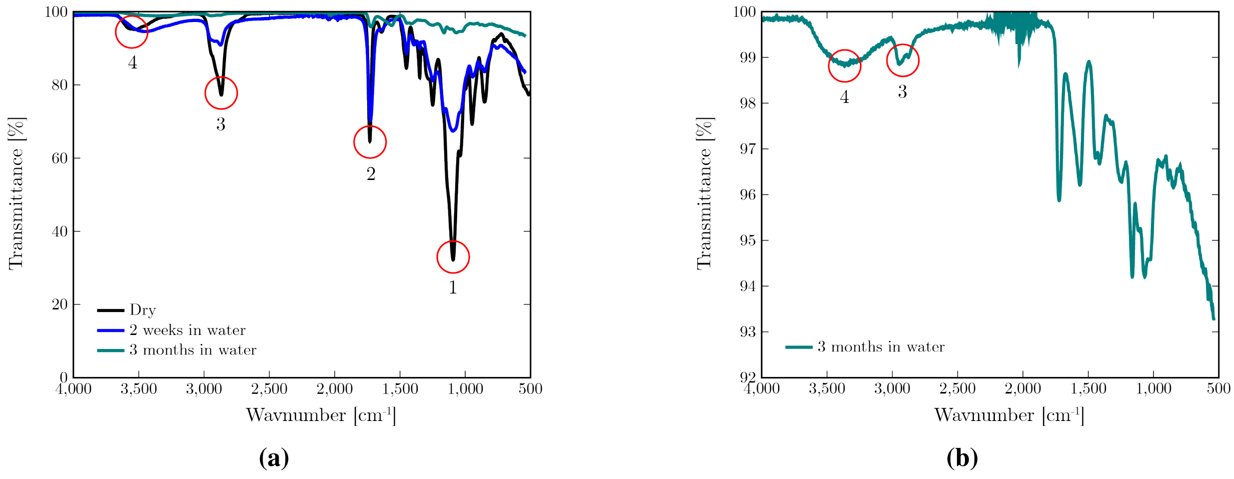

Figure S12: (a) IR spectrum of dry PEGDA (black line) and PEGDA stored in water for 2 weeks (blue line), and 3 months (teal line). (b) Zoomed-in IR spectrum of the PEGDA polymer stored in water for 3 months.

There are four peaks of interest in Figure S12. Peak (1) is a strong sharp peak in 1092 cm^{-1}, which corresponds to the stretching of the C-O ether bond of the polyethylene glycol. This peak is probably overlapping with the stretching of the C-O ester bond of the acrylate in 1100-1200 cm^{-1}. Peak (2) in 1730 cm^{-1} corresponds to the stretching of the C=O ester bond. Peak (3) in 2870 cm^{-1} corresponds to the stretching of the C-H bond of the $C=CH_2$ group. Peak (4) is a weak broad peak in 3400-3500 cm^{-1}, which corresponds to the stretching of the O-H bond in alcohol.

As the storage time in water increases, the relative intensity of the peaks corresponding to the ester and methyl groups decrease (peaks (1)-(3)), while the relative intensity of the peak corresponding to the alcohol group (peak (4)), increases. This can be explained based on the hydrolysis reaction of PEGDA in water (Figure S13). The ester bonds between the ethylene glycol and methacrylate is hydrolysed, producing acrylic acid, which is miscible with water, leaving behind only polyethylene glycol.

This is clear from the zoomed-in IR spectrum of the PEGDA polymer stored in water for 3 months (Figure S12(b)). Although the intensity of the spectrum is lower than that of the other samples, the relative intensity of the O-H peak observed in 3360 cm^{-1} (peak (4)) is comparable with that of the C-H peak observed in 2940 cm^{-1} (peak (3)), in contrast with the dry sample. This further validates the hydrolysis hypothesis, as the methyl group is removed by the sample through

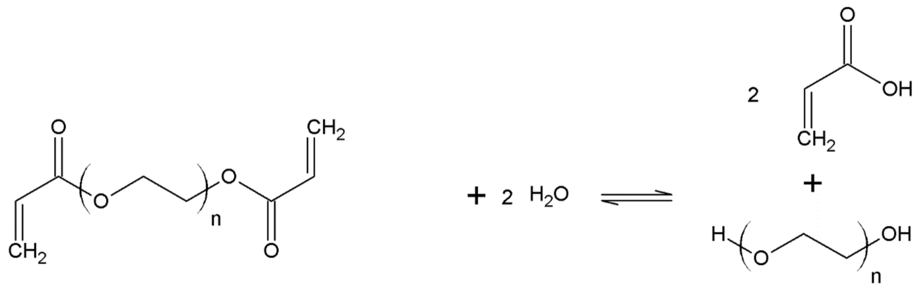

Figure S13: The hydrolysis mechanism of PEGDA, producing acrylic acid and polyethylene glycol.

acrylic acid, leaving behind the alcohol group of the polyethylene glycol.

The FTIR analysis suggests no significant PEGDA hydrolysis during permeability and separation experiments including the pre-treatment step in water. It further paves the way to study other materials such as polyethylene glycol diacrylamide (PEGDAA), which exhibits a more robust performance under water environments. In PEGDAA, the ester bond is replaced with an amide bond, preventing hydrolysis.[12]

Separation experiments

Permeate images

The images of the permeates obtained from the 3D-printed and commercial membranes after separating diluted O/W emulsions (SDS50 and SDS100) are shown in Figure S14 and Figure S15.

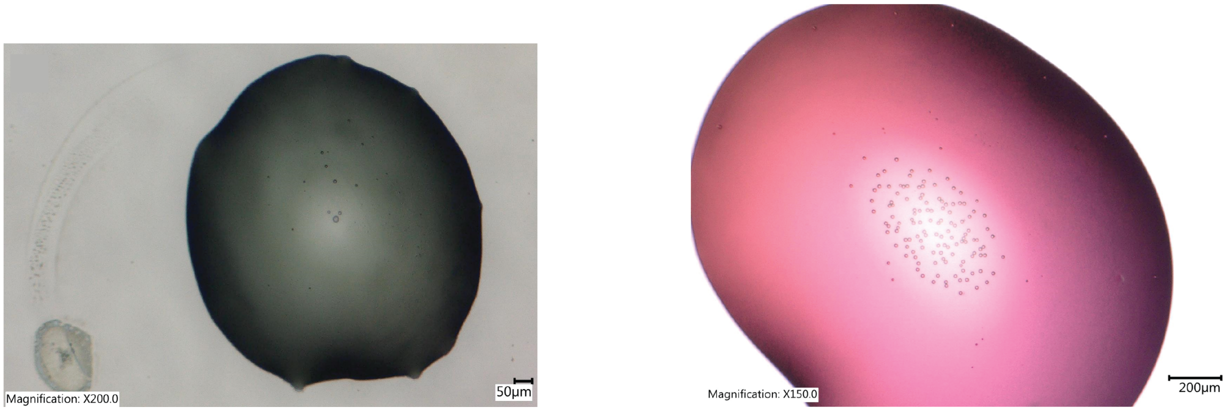

(a) **(b)**

Figure S14: Microscopy images of the permeates through 3D-printed membranes from diluted O/W emulsions: (a) SDS50 and (b) SDS100.

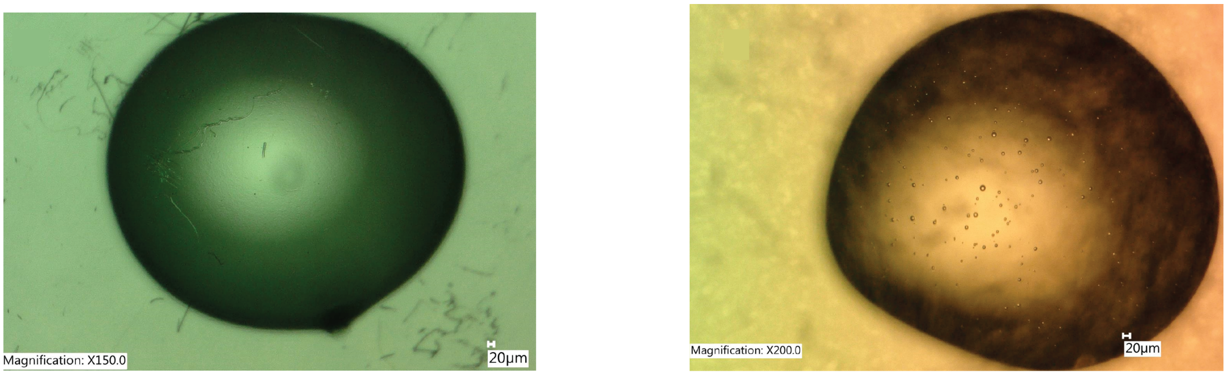

(a) (b)

Figure S15: Microscopy images of the permeates through commercial PTFE membranes from diluted O/W emulsions: (a) SDS50 and (b) SDS100.

Commercial membrane showed 100% rejection of both emulsions. The 3D-printed membranes showed 100% rejection of O/W emulsions with larger droplet size distribution (SDS50) and 90% rejection of the emulsion with smaller droplet size distribution (SDS100). It is worth noting that the droplets observed in Figure S14(a) and Figure S15(b) are air bubbles as the size do not match with the corresponding oil droplet size distributions.

Simulation results

Pore deformation

The simulation of the constrained membrane showed a significant reduction in membrane pore diameter upon pressurization. The cumulative distribution of the relative changes in pore radius at the inlet and outlet of the geometry (Figure 10 in the main text) is shown in Figure S16, which demonstrates the range of possible pore contraction depending on the model parameters.

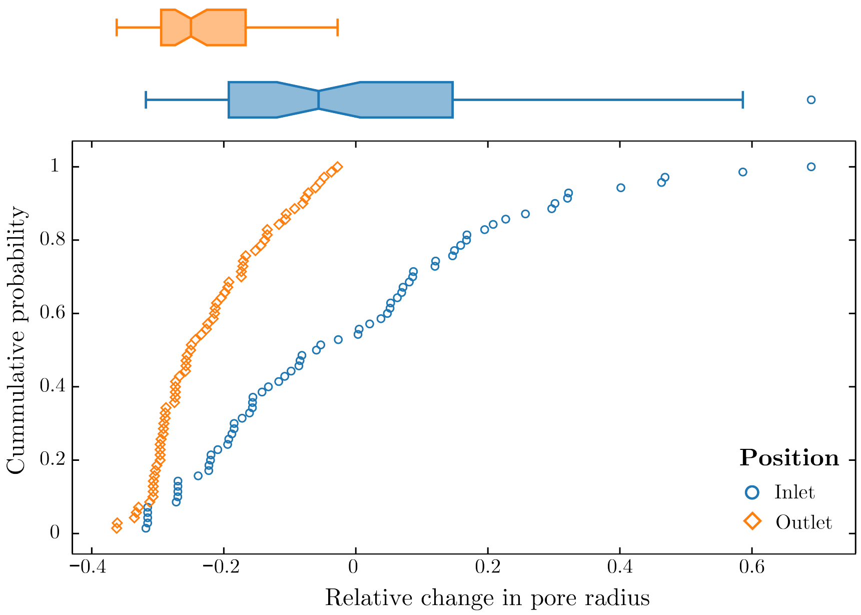

Figure S16: Cumulative distribution of the relative change in pore radius at the inlet and outlet of the geometry shown in Figure 10 in the main text.

References

(1) Schroeder, D. J. *Astronomical optics*; Elsevier, 1999.

(2) Van Der Laan, H. L.; Burns, M. A.; Scott, T. F. Volumetric photopolymerization confinement through dual-wavelength photoinitiation and photoinhibition. *ACS Macro Letters* **2019**, *8*, 899–904.

(3) Achilias, D. S.; Siafaka, P. I. Polymerization kinetics of poly (2-hydroxyethyl methacrylate) hydrogels and nanocomposite materials. *Processes* **2017**, *5*, 21.

(4) Bottou, L. *Neural networks: Tricks of the trade*; Springer, 2012; pp 421–436.

(5) Mulder, D. J.; van Vliet, W. A.; Laagland, M.; Narayanana, A.; Kostenko, A. Rapid micro-prototyping by single-photon two-wavelength volumetric lithography. Laser 3D Manufacturing X. 2023; p 1241203.

(6) Kostenko, A.; Narayanan, A. Multi-wavelength volumetric lithography. Advanced Fabrication Technologies for Micro/Nano Optics and Photonics XV. 2022; p PC120120I.

(7) Schindelin, J.; Arganda-Carreras, I.; Frise, E.; Kaynig, V.; Longair, M.; Pietzsch, T.; Preibisch, S.; Rueden, C.; Saalfeld, S.; Schmid, B.; others Fiji: an open-source platform for biological-image analysis. *Nature methods* **2012**, *9*, 676–682.

(8) Josyula, T.; Kumar Malla, L.; Thomas, T. M.; Kalichetty, S. S.; Sinha Mahapatra, P.; Pattamatta, A. Fundamentals and Applications of Surface Wetting. *Langmuir* **2024**, *40*, 8293–8326.

(9) Hagen, G. Über die bewegung des wassers in engen zylindrischen rohren. *Annalen der Physik und Chemie* **1839**, *46*, 423–442.

(10) Poiseuille, J. L. Recherches expérimentales sur le mouvement des liquides dans les tubes de très-petits diamètres. *Comptes rendus de l'Académie des sciences* **1840**, *11*, 961–967, 1041–1048.

(11) Darcy, H. *Les fontaines publiques de la ville de Dijon*; V. Dalmont, 1856; Vol. 2.

(12) Browning, M.; Cereceres, S.; Luong, P.; Cosgriff-Hernandez, E. Determination of the in vivo degradation mechanism of PEGDA hydrogels. *Journal of Biomedical Materials Research Part A* **2014**, *102*, 4244–4251.
